# Design and Biocompatibility of Biodegradable Poly(octamethylene suberate) Nanoparticles to Treat Skin Diseases

**DOI:** 10.3390/pharmaceutics16060753

**Published:** 2024-06-03

**Authors:** Dragana P. C. de Barros, Luís P. Fonseca, Luís G. Gonçalves, Diogo S. Serrano, Abel Oliva

**Affiliations:** 1 Instituto de Tecnologia Química e Biológica António Xavier, Universidad Nova de Lisboa, 2780-157 Oeiras, Portugal; lgafeira@itqb.unl.pt (L.G.G.); oliva@itqb.unl.pt (A.O.); 2 Department of Bioengineering, Institute for Bioengineering and Biosciences, Instituto Superior Técnico, Universidad de Lisboa, Avenida Rovisco Pais, 1049-001 Lisboa, Portugal; diogosserrano@gmail.com

**Keywords:** aliphatic polyesters, poly(octamethylene suberate) nanoparticle, dermal delivery, therapeutic compounds, biocompatibility

## Abstract

Biodegradable aliphatic polyester formulations as carriers for topical drug delivery show the potential to encapsulate structurally different therapeutic compounds. Poly(octamethylene suberate) (POS) nanoparticles (POS-NPs) were used as a matrix to encapsulate four therapeutic molecules used to treat skin disorders: caffeine (CF), quercetin (QR), hydrocortisone (HC), and adapalene (AD). Hydrophobicity and chemical structure of bioactive compounds (BCs) influenced the physicochemical stability of drug-loaded nanoparticles. The particle size of drug-loaded nanoparticles was between 254.9 nm for the CF-POS-NP and 1291.3 for QR-POS-NP. Particles had a negative charge from −27.6 mV (QR) to −49.2 mV (HC). Drug loading content for all BC-POS-NPs varies between 36.11 ± 1.48% (CF-POS-NP) and 66.66 ± 4.87% (AD-POS-NP), and their entrapment efficiency is relatively high (28.30 ± 1.81% and 99.95 ± 0.04%, respectively). Calorimetric analysis showed the appearance of polymorphism for AD- and HC-loaded systems and the drug’s complete solubilisation into all nanoparticle formulations. FTIR and NMR spectra showed apparent drug incorporation into the polymer matrix of NPs. The encapsulation of BCs enhanced the antioxidative effect. The prepared POS nanoparticles’ cytotoxicity was studied using two dermal cell lines, keratinocyte (HaCaT) cells and fibroblasts (HDFn). The nanoparticle cytotoxic effect was more substantial on HaCaT cell lines. A reconstructed human epidermis (RHE) was successfully used to investigate the penetration of polymeric NPs. Based on permeation and histology studies, HC-POS-NPs and CF-POS-NPs were shown not to be suitable for dermal applications with the explored drug concentrations. AD presents a high permeation rate and no toxic impact on RHE.

## 1. Introduction

The capability of bioactive compounds (BCs) to penetrate or permeate the skin barrier is essential for topical therapy of skin diseases. Nevertheless, it is also very relevant for the systemic delivery of drugs with poor peroral bioavailability [1,2]. Nanoparticles as drug delivery systems improve and control stability, activity, solubility, and bioavailability and create skin-based drug reservoirs for a sustained or stimuli-induced release [3,4]. Polymeric nanoparticles are made of biocompatible and biodegradable polymers (natural, semi-synthetic, or synthetic) with personalized physical, chemical, and biological properties. Degradability of nanoparticles, permeation capacity, and safety issues are considered of primary importance for dermal drug delivery [5,6,7,8,9]. Aliphatic polyesters (APs) are recognized for their helpful combination of biocompatibility and biodegradability and, therefore, present several advantages for pharmaceutical applications [10,11]. AP are linear polymers with ester linkages and high sensitivity to hydrolytic degradation, generating byproducts which can be eliminated from the body, resulting in the high biocompatibility of these nanoparticles [9,10,11,12]. Latterly, aliphatic polyesters have become especially interesting when chemical functionality is controlled, enabling subsequent attachment of, for example, hydrophilic drugs [12,13,14]. The essential criteria for AP for drug delivery is their biocompatibility profiles, which has enhanced interest in studying poly(alkylene dicarboxylate)s. The properties of APs are associated with the drug release rate caused by biodegradation. Polymer crystallinity, hydrophobicity, molecular weight (MW), and glass transition temperature are essential factors that influence hydrolysis [13,14,15,16]. Additionally, synthetic polymers are superior to natural polymers in terms of purity, i.e., uniformity of composition. During the formation of nanoparticles or micelles through self-assembly, hydrophobic drugs can migrate to the hydrophobic core of the polymer to form the drug-loaded carrier. These vehicles can transport the drug to the intended site through bulk erosion or stimuli-triggered release [17,18,19]. On the other hand, encapsulation of hydrophilic drugs in polymeric NPs may bring a more complex issue [13,20]. A crucial question for the investigation of nanoparticulate drug delivery carriers is the drug incorporation and the mechanism of drug release from the particles, which can occur in suspension or on the skin surface, leaving the carrier particles outside or possibly particle penetration through the skin to release the drug within the tissue.

Among these AP polymers, poly(ethylene succinate) (PES), poly(butylene succinate) (PBS), and poly(butylene succinate-co-butylene adipate) (PBSA) have been one of the most extensively used [10,21,22,23]. Although poly(alkylene dicarboxylate)s have been broadly studied [10,21,22,23], there is a lack of data on using 1,8-octane diol polyesters as nanocarriers. Nevertheless, this group of esters’ synthesis, crystallization behaviour, and good mechanical properties have been reported [23,24,25,26,27]. 

We previously demonstrated the preparation of poly(octamethylene suberate) (POS), biodegradable AP using enzymatic polymerization in a miniemulsion system and water as a reaction media using Polymer-5B technology [23,24,25]. The impact of polymer degradation products on cells and tissue is crucial for applying biodegradable polymers [28]. POS is based on a 1,8-octane diol and moderate acid monomer, suberic acid, with a low melting temperature (60–70 °C). Kand et al. [29], based on in vivo studies with mice, reported the potential of suberic acid as a skin antiphotoaging agent. Additionally, suberic acid used in the preparation of reduction-sensitive micelles affect their cellular uptake and has potential application in delivery of anticancer drugs [30].

The low melting point of POS polymers, 61.1 °C [23], allow its easier processability for biomedical applications in comparison with some other APs (e.g., PLA and PGA melt at 150 and 225 °C) [31]. This work used the oil-in-water one-step miniemulsion process without using organic solvents to generate POS nanocarriers to encapsulate bioactive compound models used to treat skin disorders [28,32,33]. Additionally, the molecular weight of synthesised POS [23] indicates slow degradation, which is suitable for the long-term and controlled release of encapsulated drugs and lowers the negative impact on the tissue. Fast polymer degradation occurs in the accumulation of acidic breakdown products that accelerate the rate of hydrolysis, and a solid or middle acidic core can be detrimental to host cells’ proliferation (necrosis) or promote infection in the surrounding tissues. To the author’s knowledge, there is no report of POS used for NP production and applications. 

**Figure 1:**
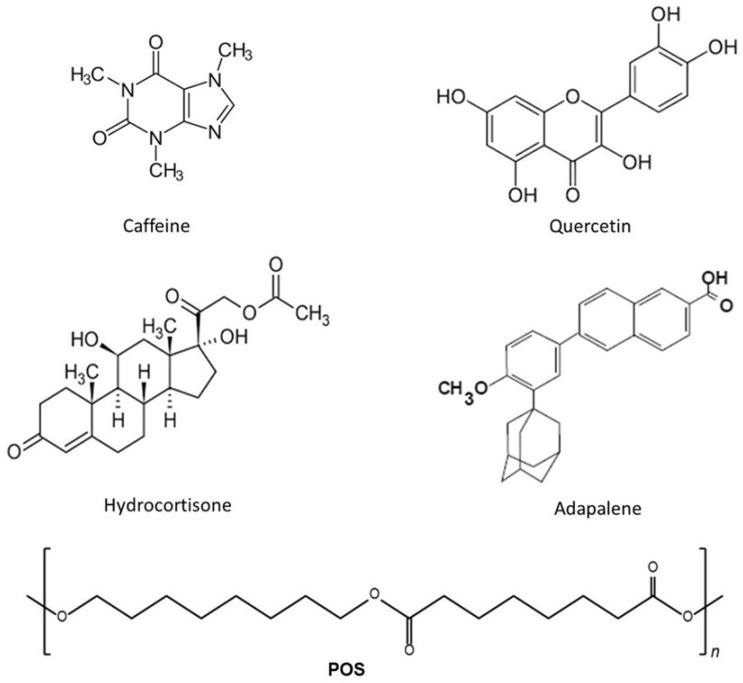
Chemical structures of caffeine (CF, CAS 58-08-2), hydrocortisone (HC, CAS 50-23-7), quercetin (QR, CAS 117-39-5), and adapalene (AD, CAS 106685-40-9) and POS (Mw = 13,806 g mol^−1^).

Quercetin (QR), caffeine (CF), hydrocortisone (HC), and adapalene (AD) (Figure 1) with reported therapeutic effects on skin disorders were chosen based on their origins (from plant extract (CF, QR) or synthetic (HC, AD)), different physicochemical characteristics (e.g., hydrophobicity), and therapeutic effect (e.g., antioxidant, anti-inflammatory, antiageing), which were used to analyse the capability of POS-NPs to capture drugs for topical applications. 

Quercetin (QR, 3,5,7,30,40-pentahydroxyflavone) is one of the most versatile polyphenolic compounds with vast therapeutic potential. QR achieved GRAS (Generally Recognised As Safe) status by the United States Food and Drug Administration (FDA) [34,35,36]. Multiple hydroxyl groups in quercetin are likely responsible for its biological activity and reactivity [37]. Therapeutic applications of QR on the skin are due to its antioxidant, anti-inflammatory, anticancer, antiaging, and antibacterial properties. Quercetin as a molecule is a model for a lipophilic drug with octanol–water partition coefficient log *p* = 1.82 [38]. 

Caffeine (CF) (1,3,7-trimethylpurine-2,6-dione) is a natural methyl xanthine alkaloid administered cutaneously for several applications to treat skin disorders, such as cellulite, to slow the photoageing process of the skin by absorbing UV radiation as topical formulations, and to promote microcirculation of the skin through its antioxidant properties [39,40,41,42]. Thus, caffeine use in consumer products, including cosmetics, continues to increase. Caffeine is recommended as a test substance by the OECD because it has been studied extensively in vitro and in vivo in dermal toxicity studies, and presents very interesting properties such as antioxidant properties, unusual solubility behaviour in non-aqueous solvents, and forms aggregates in aqueous solutions. It is a relatively lipophilic and water-soluble molecule, as indicated by its LogP of −0.07 [39,40,41,42].

Hydrocortisone (HC) is a class V–VII, low-to-intermediate-potency corticosteroid primarily used to treat atopic dermatitis due to its antiallergic and anti-inflammatory effects. Aside from the frequent use of topical corticosteroids in the therapy of psoriasis and atopic dermatitis, they can cause undesirable local side effects like skin atrophy, rosacea, erythema, and skin thinning. Encapsulation of HC in nanocarriers is beneficial in avoiding these side effects. HC is a hydrophobic molecule with only two hydrophilic contributions on the steroid rings. The LogP value is 1.61 [43,44,45,46].

Retinoids have been used as a favourite therapy for treating acne and reported antioxidant effects, but they have many limitations [47]. Adapalene (AD) is a third-generation retinoid approved by the FDA for treating acne with anti-inflammatory, keratolytic, and antiseborrheic effects. By structure, AD is a naphthoic acid in which the phenolic hydroxy group has been converted to its methyl ether [22,47,48]. Additionally, AD has been used in combination with other drugs (e.g., topical antioxidants) to treat different skin disorders such as photoaging and depigmentation effects [49]. During the AD treatment, some local side effects can include erythema, dryness, peeling, burning, and itching [22,47,48,49].

The present work presents a stable POS-NP that can encapsulate bioactive compounds with different hydrophobicity levels to treat skin disorders. The physicochemical characterisation of empty POS-NPs, loaded BC-POS-NPs, and drug incorporation into nanocarrier was analyzed. For the application of skin care products, antioxidant activity is one of the most critical factors for efficient skin protection against UV radiation or other environmental factors [50]. A DPPH assay was used to measure the hydrogen-donating activity of BCs and BC-POS-NPs to explore whether encapsulation influences the antioxidant capacity of chosen pure bioactive compounds. QR and CF, plant-based BCs, have already reported antioxidant activity, while AD and HC’s antioxidant activity was mostly reported in conjugation with other antioxidants encapsulated in nanocarriers. Biocompatibility of the systems towards two dermal cell lines (keratinocytes and fibroblasts) and permeation studies on reconstructed human epidermis were performed to investigate the proposed polymeric nanoparticles’ safety and permeation efficiency. The BC-POS-NP formulation (BC concentration, polymer concentration, type, and surfactant concentration) was chosen based on the literature and previous studies with other types of nanocarriers [51,52].

## 2. Materials and Methods

### 2.1. Materials

Poly(octamethylene suberate) (POS) was synthetized by the authors in the Institute for Bioengineering and Biosciences (Lisbon, Portugal) as previously explained [20,21,22]. Span 80 (Sorbitan monooleate, HLB 4.7) and caffeine (CF) (99.7%) were bought from Alfa Aesar (Haverhill, MA, USA). Hydrocortisone (HC) (98%) was purchased from Thermo-Fisher Scientific (Waltham, MA, USA), and Quercetin (≥95%) from SCB Santa Cruz Biotechnology Inc. (San Juan, CA, USA). Milli-Q grade water was used to prepare miniemulsion. Cell lines: human immortalized keratinocytes (HaCaT), human dermal fibroblasts, neonatal (HDFn), the cell media reagents, DMEM (Dulbecco’s modified Eagle’s medium), fetal bovine serum (FBS), trypsin 0.25%, Pen Strep (10,000 U/mL penicillin, 10 µg/mL streptomycin), Trypsin-EDTA (0.25%), phenol red, phosphate-buffered saline (PBS) 1X Solution pH 7.4, and the reagent MTT (3-(4,5-dimethylthiazol-2-yl)-2,5-diphenyltetrazolium bromide) were purchased from Gibco, Thermo-Fisher Scientific (Waltham, MA, USA). Trifluoroacetic acid (TFA) suitable for HPLC ≥99.0% was pursued from Sigma Aldrich (St. Louis, MO, USA). Acetonitrile, absolute ethanol, and methanol were of analytic grade (Alfa Aeser, Haverhill, MA, USA).

### 2.2. Polymer Molecular Weight Assay by Size-Exclusion Chromatography (SEC)

**Figure 2:**
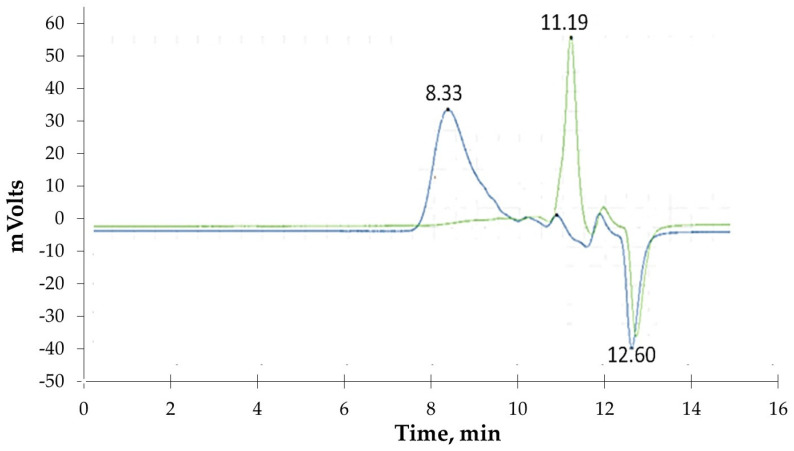
SEC spectra were obtained from the synthesis of poly(octylmethylene suberate) (POS) for the sample at time zero (green line) with an average Mw of 180 g mol^−1^ for residence time (RT) = 11.2 min, corresponding to a mixture of octanedioic acid and 1,8-octanediol for equimolar monomer concentrations (0.5 M) and sample after 48 h of polycondensation (blue line) at 55 °C with synthesised polyesters corresponding to residence time of 8.33 min (average Mw = 13,806 g mol^−1^). The peak RT of 12.6 min corresponds to water in the partially air-dried samples.

The molecular weights of the generated polymers were analysed before particle production by size-exclusion chromatography (SEC), as previously described [23]. Briefly, the average molecular weight (Mw) of the polyesters was determined by SEC using a high-performance liquid chromatography (LaChrom HPLC) device equipped with a refractive index detector (Merck LaChrom RI Detector L-7490, Darmstadt, Germany) and a polystyrene/polydivinylbenzene column (ResiPore Agilent, Santa Clara, CA, USA). The elution solvent was THF at a flow rate of 0.5 mL min^−1^ at 40 °C. The calibration curve with molecular weight polystyrene standards between 660 and 482,400 g mol^−1^ was determined. The samples were centrifuged for 10 min at room temperature (25 °C) and 10,000× *g* (Eppendorf Centrifuge 5415 D); the water supernatant was removed. The standards and dried samples were solubilised in THF, submitted to a thermal shock at 40° C for 5 min, and then centrifuged at room temperature before SEC analysis. The experimental error associated with repeated injection of the same polymer sample was inferior to 3%. The monomers’ (octanedioic acid and 1,8-octanediol) peak present a residence time (RT) of 11.2 min, while the synthesised polyesters (POS) correspond to an RT = 8.3 min (average Mw = 13,806 g mol^−1^) after 48 h (see Figure 2).

### 2.3. Fabrication of POS Nanoparticles (POS-NP and BC-POS-NP) 

#### 2.3.1. POS-NP and BC-POS-NP Production

POS nanoparticles without (POS-NP) and with bioactive compounds (BC-POS-NP) were prepared by the oil-in-water one-step miniemulsion methodology using an ultrasonication step as previously reported [28,32,33,52]. Four different BCs (CF, HC, QR, and AD) were selected for this study based on their hydrophilic–lipophilic chemical structure and potential to treat skin disorders. 

First, the polymer was heated to 70 °C, approximately 10 °C above its melting point. Meanwhile, the water solution of Span 80 (2 wt%) was heated to the same temperature. Once the desired temperature was reached, the heated polymer and aqueous phases were mixed on the same temperature, and the resulting emulsion was stirred for 2 h at 300 rpm. Then, the complete homogenisation with a Sonifier (Branson 450D, Danbury, CT, USA) was done for 10 min (10 s on/5 s off, 60% amplitude). The nanoemulsion was then cooled to room temperature and stored for 24 h before being characterised.

For the BC-POS-NP formulations, QR, AD, and HC were added in the oil (polymer) phase, while CF was added in the aqueous phase. Table 1 provides the detailed composition of each POS-NP formulation. The resultant nanoemulsion was then cooled to room temperature and stored to be characterised after 24 h. Each NP formulation was prepared and tested in triplicate.

#### 2.3.2. Lyophilisation of BC-POS-NPs

Samples of BC-POS-NP were freeze-dried under vacuum using a lyophiliser Ed-wards Micromodul (BOC Ltd., Crawley, UK). A cooling rate of 1 °C min^−1^ was used to precool the sample from room temperature to −50 °C, and the sample was maintained at −50 °C for 24 h [52].

### 2.4. Nanoparticle Physicochemical Characterisation

#### 2.4.1. Size, PDI, and Z-Potential of POS-NP and BC-POS-NP

The hydrodynamic diameter of the POS-NP particles and polydispersity index (PDI) were detected by dynamic light scattering at 25 °C and a scattering angle of 173° (Zetasizer^®^Nano ZS, Malvern PCS Instruments, Malvern, UK) 24 h after preparation from original nanoemulsions produced in Section 2.3.1. The samples were added to a cuvette without dilution before the measurement. Particle sizes and PDIs are given as the average of three measurements. The equipment’s controlling software performed data processing, and the particle size data were evaluated using the intensity distribution.

The zeta (Z)-potential of BC-POS-NP was measured by electrophoretic mobility using the same equipment. The analyses were conducted at 25 °C, and the samples were diluted with Milli-Q water (1:10, *v*/*v*). The reported values are the mean ± standard deviation (SD) of at least three different batches of each BC-POS-NP formulation.

#### 2.4.2. Determination of Entrapment Efficiency (EE) and Drug-Loading Capacity (DL)

The EE (%) and DL (%) of BC-POS-NPs loaded with CF, QR, HC, and AD were cal-culated by measuring the concentration of each bioactive compound in the dispersion medium of the nanoparticles using a reverse-phase high-performance liquid chromatography (RP-HPLC) method.

The unencapsulated BCs were separated from the nanoparticle dispersion by centrifugation at 17,000 rpm for 1.5 h at 4 °C. The supernatants with no encapsulated bioactive substance were collected and analysed via HPLC. The BC content in the supernatant was detected by HPLC Waters Alliance 2695 HPLC Separations Module (Dublin, Ireland) using an Avantor^®^ ACE 5 C18 (100 Å, 5 m, 250 mm × 4.6 mm) column. The supernatant of each active substance was dissolved in methanol for analysis.

The mobile phase for QR detection consisted of acetonitrile and 0.1% TFA (35:65, *v*/*v*). A flow rate of 1.0 mL min^−1^ was carried out, and the signal was detected at 370 nm with the column temperature maintained at 30 °C [52]. The operating conditions for the HC analysis were set based on the methodology described by Adi-Dako et al. [53] using a mobile phase of composition methanol: water: acetic acid (60:30:10, *v*/*v*/*v*) at a flow rate of 1.0 mL/min at a wavelength of 254 nm and the column was kept at ambient temperature. CF concentration was measured using the mobile phase (water: methanol at a ratio of 60:40 % *v*/*v*), the column temperature was kept at 40 °C, a flow rate of 1 mL/min, and a wavelength of 275 nm was adopted [42]. A mixture of acetonitrile, tetrahydrofuran (THF), and water containing 0.1% acetic acid (25:50:25) was used as a mobile phase in an isocratic elution with a constant flow rate of 0.8 mL/min, with the UV detector set at 270 nm for AD detection [54]. The injection volume of all samples was 50 μL, and retention times were 2.96 min, 4.36 min, 6.48 min, and 7.05 min for CF, HC, QR, and AD, respectively. *EE* (%)and *DL* (%) of bioactive compounds in BC-POS-NP were calculated according to the following equations:

$$EE,\%=\frac{Wt-Wf}{Wt}*100$$

$$DL,\%=\frac{Wt-Wf}{Wt-Wf+Wp}*100$$

where *Wt* is the total mass of BC added to the whole system, *Wf* is the mass of free BC determined in the dispersion medium, and *Wp* is the mass of the polymer in NP formulations. The reported results are the mean ± SD of at least three different batches of each BC-POS-NP formulation.

### 2.5. BCs Incorporation Studies

#### 2.5.1. Crystallinity Studies by Differential Scanning Calorimetry (DSC)

Differential scanning calorimetry (DSC) analysis was performed to analyse the crystalline state of POS-NP and POS-NP-BC with 0.5 wt% BC. The thermograms were record-ed using a DSC Q200 F3 (TA Instruments Inc., New Castle, DE, USA). A nitrogen purge provided an inert gas atmosphere within the DSC cell at a 50 mL min^−1^ flow rate. A constant heating or cooling rate of 5 °C min^−1^ was applied. Approximately 3–5 mg of dried POS-NP and BC-POS-NP samples were hermetically sealed into standard aluminium pans. As a reference, an empty pan was used. The samples were equilibrated at 0 °C and then submitted to a heating cycle from 0 to 100 °C and a cooling cycle from 100 to 0 °C. For AD-POS-NP, the temperature regime is from −20 to 100 °C (heating) and from 100 to −20 °C (cooling). All samples were scanned twice; the first run was to level the bottom of heterogeneous polymer samples on the surface of the DSC pan, and the second run was used for DSC parameter calculations [55]. Afterwards, all samples were submitted to a heating cycle from 0 to 400 °C. The melting temperatures (Tm, °C), cooling temperature (Tc, °C), glass transition temperature for melting and crystallisation (Tgm and Tgc, °C), and enthalpies (ΔH, J g^−1^) were evaluated using the TA Universal Analysis 2000 (v4.5.0.5) software ((TA Instruments Inc., New Castle, DE, USA). 

#### 2.5.2. Fourier Transform Infrared (FTIR) Analysis

FTIR spectra of pure compounds, POS-NP, and BC-POS-NP were obtained at ambient temperature using a Bruker IFS 66/S FTIR Spectrometer (Bruker Optics, Ettlingen, Germany) in the range of 520–4000 cm^−1^. The spectra were taken as the average of 32 scans at a resolution of 4 cm^−1^. The experiments were performed in triplicate.

#### 2.5.3. Nuclear Magnetic Resonance (NMR) Analysis

The molecular structures of the synthesised POS-NPs and BC-POS-NPs were con-firmed by ^1^H-NMR. ^1^H-NMR spectra were obtained on an Avance II+ 400 Bruker NMR nuclear magnetic resonance spectrometer equipped with BBFO-z X-H-D g, a 5 mm probe, at 25 °C. The solvent used was deuterated chloroform (CDCl3-d) (99.8%, Cambridge Isotope Laboratories) for AD-POS-NPs, HC-POS-NPs, and CF-POS-NPs at a concentration of 6 mg mL^−1^. For the QR-POS-NPs, deuterated DMSO (DMSO-d6; Sigma Aldrich) was used. ^1^H-NMR spectra for POS-NP were acquired in both solvents. The zg30 pulse program was used with a relaxation delay of 1 s, a sweep width of 64 K, and a total of 64 scans. The spectra were compared regarding the residual CDCl3-d peak (at 7.3 ppm) or the TMS signal (at 0.0 ppm), and the chemical shifts (d) were related in parts per million (ppm).

### 2.6. Antioxidant Activity of BC-POS-NP

The free radical scavenging (antioxidant) capacity of pure BCs (CF, HC, QR, and AD), POS-NP, and BC-POS-NP were measured by 1,1-diphenyl-2-picrylhydrazyl (DPPH) assay [56]. An amount of 200 µL of sample solution was added to 100 µL of DPPH solution (0.2 mM) prepared in absolute ethanol. The reaction mixture was incubated at 37 °C for 30 min. The absorbance was measured at 517 nm in a multiwell plate reader (SpectraMax 340PC Microplate Reader, Molecular Devices, LLC., San Jose, CA, USA) against the DPPH control solution. The radical scavenging activity was calculated using the following equation:

$$Scavenging\;activity,\;\%=\frac{Ac-As}{Ac}*100$$

where *Ac* is the absorbance of control at λ = 517 nm, and *As* is the absorbance of sample at λ = 517 nm, 30 min after incubation in the presence of antioxidant. All determinations were performed in triplicate and the results are given as the mean ± SD.

### 2.7. Biological Assays

#### 2.7.1. Cytotoxicity of BC-POS-NP

The cytotoxicity tests were performed for two dermal cell lines: immortalized human keratinocytes (HaCaT) and human dermal fibroblasts, neonatal (HDFn) [51,52]. HaCaT and HDFn were cultured in 175 cm^2^ flasks using Dulbecco’s modified Eagle’s medium (DMEM) supplemented with 10% fetal bovine serum (FBS) and 0.1% Pen Strep (10,000 U/mL penicillin, 10 µg mL^−1^ streptomycin). Cells were maintained at 37 °C in a 95% air/5% CO_2_ atmosphere and were detached with a trypsin solution (Trypsin/EDTA Solution, Gibco™). The cell lines were harvested at 80% confluence and were seeded in each well of 96-well plates at a density of 2 × 10^4^ cells/well. Cells were grown for 24 h at 37 °C in a 95% air/5% CO_2_ atmosphere to obtain subconfluence. Cells were then washed with a PBS solution and put in contact with 200 µL of each sample. Pure BC, POS-NP, and BC-POS-NP samples were diluted with DMEM medium in 1/10, 1/20, 1/30, 1/40, 1/50, and 1/100 *v*/*v* ratios. For the positive control, cells were kept in contact only with the culture medium without FBS. The cytotoxicity of the systems was evaluated by the MTT reduction assay [53]. Briefly, 10 µL of the 12 mM MTT was added to each well with the sample and incubated in a humidified 5% CO_2_–95% air atmosphere for 3 h. After this incubation period, the samples were removed from the wells, washed with PBS to remove unreacted MTT, and the formed formazan crystals dissolved in 100 mL dimethyl sulfoxide (DMSO) for 10 min at 37 °C. The absorbance was measured at 540 nm in a multiwell plate reader (SpectraMax 340PC Microplate Reader, Molecular Devices, LLC., San Jose, CA, USA). The cell viability (%) was calculated using the following equation:

$$Cell\;viability,\;\%=\frac{Absorbance\;of\;treated\;cells}{Absorbance\;of\;negative\;controle}*100$$

where the negative control was the cells incubated with DMEM medium without FBS addition. 

All experiments were performed at least in triplicate and the results are given as the mean ± SD.

#### 2.7.2. Cultivation of Reconstruction of Human Epidermis (RHE) 

Primary human epidermal keratinocytes isolated from neonatal foreskin (HEKn; Gibco) were maintained in keratinocyte growth medium (KGM) composed of EpiLife medium (Gibco), supplemented with 0.06 mM calcium and keratinocyte growth factor (HKGS, Gibco) at 37 °C and 5% carbon dioxide (CO_2_) in a humidified incubator. 

**Figure 3:**
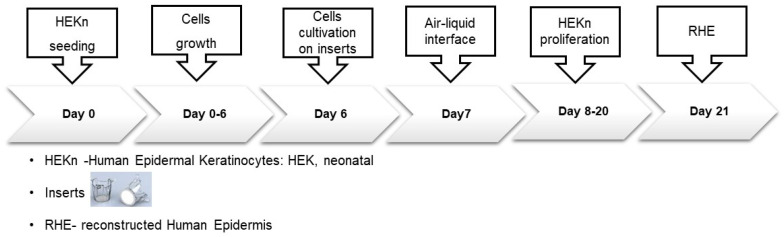
Schematic overview of RHE model generation [50].

The development of RHE on cell culture inserts (Figure 3) with polycarbonate membranes (Merck, Millipore) was performed following the procedure described by Zoio et al. [57]. In brief, the inserts were placed in six-well plates containing 2.5 mL of HEK growth medium with high calcium concentration (1.5 mM) and seeded with 3 × 10^5^ HEKns in 500 mL KGM medium. After 24 h, the models were raised to air–liquid phase. The medium was replaced by 1.5 mL KGM supplemented with 1.5 mM calcium, 50 mg mL^−1^ l-ascorbic acid 2-phosphate, and 10 ng/mL keratinocytes growth factor (KGF) (San Diego, CA, USA). This culture medium was replaced every 48 h and after 11 days at the air–liquid interface, until the RHE was morphologically fully differentiated.

#### 2.7.3. In Vitro Permeation Studies on RHE Using Franz Diffusion Cells 

In vitro permeation studies of BC-POS-NPs dispersions were performed using engineered static Franz diffusion cells designed and constructed at the Biomolecular Diagnostic Laboratory at Instituto de Tecnologia Química e Biológica da Universidade Nova de Lisboa (ITQB NOVA, Oeiras, Portugal). The diffusion cells have an effective diffusional area of 2.54 cm^2^ and a chamber capacity of 13.9 mL to accommodate the inserts used to support the RHE. 

The study was conducted according to the conditions established in the OECD Guideline 428 (2004) [58]. The inserts supporting the formed RHE were mounted on the receptor compartment of the Franz diffusion cells. The diffusion cells were filled with the receptor medium and were left to hydrate for 1 h in a thermostatic water bath at 37 °C ± 0.5 °C under constant stirring at 250 rpm. 

The receptor medium for QR-POS-NP consisted of a solution of absolute ethanol: distilled water (35:65 *v*/*v*) with sink conditions. The receptor medium for AD-POS-NP consisted of a mixture of citrate buffer (pH 4.0) and THF (4:1 *v*/*v*) containing 2 wt% SDS [47,53]. For the systems CF-POS-NP and HC-POS-NP, the receptor medium was phosphate buffer pH 7.4 [42,59].

After the hydration (1 h), 500 μL of the BC-POS-NP dispersion were added into the donor compartment. Aliquots of 1 mL were withdrawn at established time intervals (0, 5, 15, 30, 45, 60 min and 1, 2, 3, 4, 5, 6, 7, 8, and 24 h up to 24 h) and replaced with the same volume of fresh receptor medium. The results were expressed as cumulative amount (Qt) of BCs permeating the RHE membranes (Equation (1)):

(1) $$Qt=\left(Cn*Vs*\sum_{i-1}^{n-1}Ci*Vr\right)/S$$

where (*Cn*) is the bioactive substance concentration (μg/mL) in the receptor medium at each sampling time “*n*”, *Ci* is the bioactive substance concentration (μgmL^−1^) at time “i”, *Vr* (mL) is the volume of the receiver solution, *Vs* (mL) is the volume of the sample, and *S* characterises the sectional area of tissue (cm^2^).

The cumulative amount (μgcm^−2^) of BC was plotted as a function of time, which allowed for the determination of the permeation rate at steady state (J, μ/cm^2^ h) from the slope of the linear regression. 

The permeability coefficient (*P*, ×10^−4^ cm h^−1^) was calculated by Equation (2):

(2) P=J/C0

where *J* is the steady state flux (μgcm^−2^ h) and *C0* is the initial drug concentration in the donor side (μg cm^−3^), which correspond to the concentrations of BC loaded in the BC-POS-NPs that were quantified by HPLC following the procedure described in Section 2.7. The experiments were performed in triplicate. All samples were collected in vials and kept at −20 °C until analyzed by HPLC. All experiments were performed at least in triplicate and the results are given as the mean ± SD.

#### 2.7.4. Histological Analyses

For the histological analysis of the permeation studies, the inserts were removed from the Franz diffusion cells and the surface supporting the RHE was washed with 1 mL PBS, pH 7.4 (1X) to remove residual POS-NP and BC-POS-NPs. The samples were fixed immediately after being taken out of culture in 10% neutral buffered formalin (Sigma-Aldrich) for a minimum of 24 h at room temperature. 

Skin sections (5 mm thick) were mounted on slides for histological analysis. Tissue sections were conventionally stained with hematoxylin–eosin staining to allow a standard morphological analysis of the RHE. Images were obtained using the Nikon Eclipse TE2000-S fluorescence microscope (Nikon instruments, Melville, NY, USA) and analyzed with the ImageJ Software version 1.53q.

### 2.8. Statistical Analysis

Statistical analysis of variance for particle size, PDI, and ZP was performed with Microsoft Excel 2013 software (Microsoft, Redmond, WA, USA) by normal distribution using a significance level of α = 0.05. All quantitative results were obtained from triplicate samples. Every data point was expressed as mean ± SD. 

## 3. Results and Discussion

### 3.1. Preliminary Studies

The authors previously developed an innovative enzymatic polycondensation process for synthesising polymers using a two-step polymerization method based on the new greener polymer-5B technology [23]. The process involved combining dicarboxylic acids and dialcohols in different reaction media, including organic solvent, miniemulsion, and water. Prior to synthesising particles, it was important to test the biocompatibility of the polymers on the main dermal cell lines, namely keratinocytes (HaCaT) and fibroblasts (HDFn). Keratinocytes are the primary component of the epidermis skin layer, while fibroblasts are responsible for skin hydration and elasticity. To use nanocarriers in pharmaceutical products, it is necessary to have a detailed understanding of their efficacy, quality, and safety. Therefore, in vitro toxicity tests must be conducted to determine the biocompatibility of the synthesised POS polymers in cultured cells.

**Figure 4:**
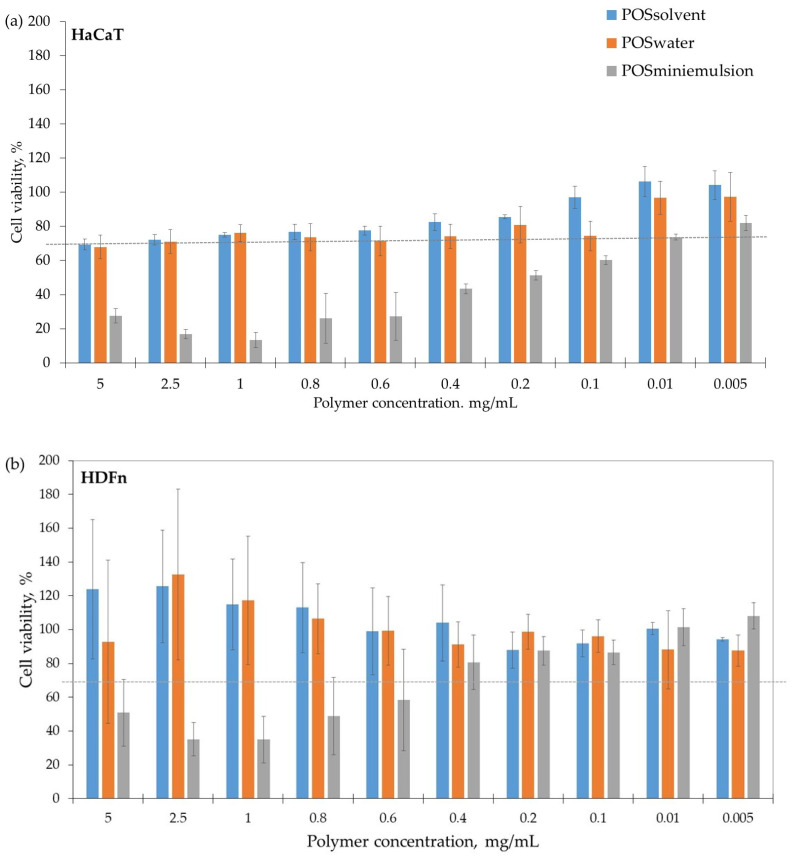
Cytotoxic effect of POSsolvent, POSwater, and POSminiemulsions on (**a**) keratinocytes (HaCaT) and (**b**) fibroblasts (HDFn); medium (DMEM) without FBS supplement was used as control.

Figure 4a,b illustrates the obtained results. Regarding ISO 10993-5:2009 [60], a reduction in cell viability by more than 30% was considered a cytotoxic effect. All formulations showed a concentration-dependent effect, with toxicity increasing proportionally to the POS concentration. POS synthesised in a miniemulsion system shows a significantly higher degree of toxicity than solvent and water as reaction media.

The presence of impurities, such as the surfactant (Triton-X100), present in traces in polymers synthesised in miniemulsion reaction media [23,25] may increase its toxic effect. Nontoxic concentrations of POS_miniemulsion_ polymer are < 0.4 mg mL^−1^ (Figure 4). At the same time, for the other two systems, there is no toxicity for both cell lines for all scales of tested concentration (0.005–5 mg/mL). Aside from the degree of purity and Mw of POS_organic solvent_ corresponding to POS_water_, the increasing sensitivity for environmentally friendly processes and the preference for “green” technology, POS_water_ (forward named as POS; average Mw = 13,806 g mol^−1^, Figure 2) was used for nanoparticles synthesis and the encapsulation studies. 

### 3.2. Physiochemical Characterisation of POS-NPs and BC-POS-NPs

#### 3.2.1. Physicochemical Properties of BC-POS-NP Formulations

**Figure 5:**
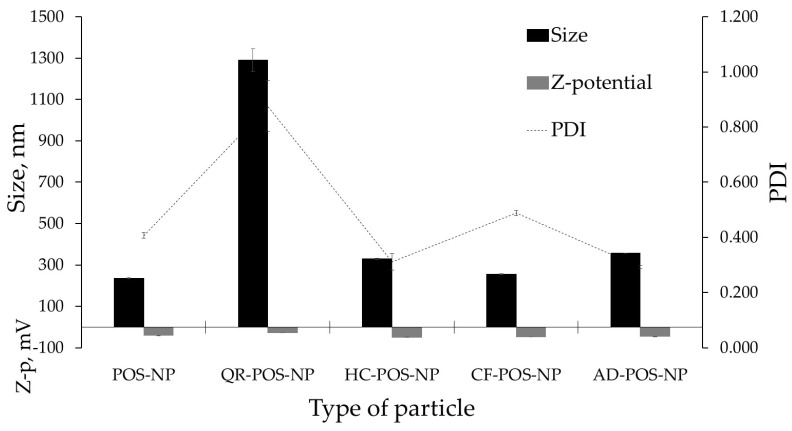
Physicochemical characteristics for POS-NP and BC-POS-NPs; BC—bioactive compound; QR—quercetin, HC—hydrocortisone; CF—caffeine; AD—adapalene; NP—nanoparticle; 0.5 wt% of BC; Span 80.

The effects of drug loading on the mean particle size and physical stability of the free POS-NP and four drug-loaded BC-POS-NP systems (BC: CF, HC, QR, AD) are presented in Figure 5.

As the objective of this study was to determine the capability of POS to encapsulate structurally different bioactive compounds in this step, only structural influence on the physicochemical stability of the system was analyzed. Therefore, the BC concentration (0.5 wt%), type, and surfactant concentration (Span 80, 2%, *w*/*w*) were chosen based on the literature review and were kept constant [52]. The particle size was statistically significant and ranged between 236.6 ± 7.2 nm for no encapsulated POS-NP (PDI 0.407 ± 0.13) and 357.7 ± 16.7 nm for AD-POS-NP (PDI 0.292 ± 0.021). A considerable increase in the particle size to the micro range (1291.3 ± 54.5 nm), also followed by a PDI increase (PDI 0.876 ± 0.49), was found for the QR encapsulation. As a PDI determines the degree of heterogeneity of the size distribution of particles, the QR-POS-NP system indicates possible agglomeration phenomena leading to large particle size. 

The mean particle size for HC-POS-NP was 330.8 ± 10.4 nm, with a PDI value of 0.312 ± 0.33.

The mean particle size of 254.9 ± 2.9 nm for the CF-POS-NP, with a PDI value of 0.488 ± 0.21 nm, was close to POS-NP size and PDI values. A PDI of 0.3 and below for nanocarriers suggests a homogenous population.

Zeta potential (ZP) is an important variable in the physical stability of dispersions, and higher values of ZP (>|30|) tend to stabilize the nanoparticle dispersion and avoid aggregation phenomena due to electrostatic repulsions between particles. The Z-potential values for POS-NP were −40.9 ± 4.3 mV. Encapsulation of BC increases the negative value of particle charge for CF-, HC-, and AD-POS-NPs, by 14, 17, and 13%, respectively. However, for QR-POS-NP, the Z-potential change to −27.6 ± 5.5 mV confirmed the low stability of this system, observed by very large mean size and PDI value. The presence of multiple hydroxyl groups placed at the C 3-, 3′-, 4-, 5-, and 7-positions in QR may be responsible for interaction with the polymer and water media, leading to the generation of larger particles and lower Z-potential.

As the nature of the bioactive compound influenced the physicochemical stability of BC-POS-NP systems, it was essential to investigate the encapsulation efficiency (EE, %) and drug loading (DL, %) of these systems.

#### 3.2.2. Entrapment Efficiency (EE, %) and Drug Loading (DL, %) Capacity

Table 2 shows the results of EE and DL of all BC-POS-NPs. The concentration of BC in all cases was 0.5 wt%. The type of bioactive compound was found to have a significant impact on the degree of drug entrapment.

As shown in the results, the degree of encapsulation and drug loading decreases as the BC’s hydrophobicity decreases: AD = QR > HC > CF. AD-POS-NP and QR-POS-NP exhibited the highest entrapment efficiencies among all the prepared systems, with a maximum EE of 99.5 ± 0.04 (%) for AD-POS-NP and a minimum EE of 28.30 ± 1.81 (%) for CF-POS-NP. DL follows the EE tendency (Table 2).

The low efficiency of CF encapsulation showed that POS-NP particles in this form are not suitable carriers for the hydrophilic compounds. On the other hand, the high efficiency of QR encapsulation may be connected to the hydrophobicity of the QR molecule. Aside from the high EE and low physicochemical stability, QR cannot be encapsulated in POS-NP under the conditions described. For example, A. Choudhary et al. [61] stated EE of 90 ± 3.30% for the QR in chitosan tripolyphosphate nanoparticles. Nevertheless, in this case, the average size of QR nanoparticles was approximately 360 nm with PDI < 0.2, indicating a homogeneous and stable system. HC-POS-NP (EE, 83.09%; DL, 62.42%) and AD-POS-NP (EE, 99.95%, DL, 66.66%), regarding physicochemical stability and drug capture efficiency, showed to be promising candidates for encapsulation in POS-NPs. Malekhosseini et al. reported 79% loading efficiency of Dextran–PLGA/hydrocortisone-based nanomicelles [46]. 

Thermal calorimetric studies were performed with all systems to better understand the complexity of BC-POS-NP bonds. 

### 3.3. Thermal Characterisation by Differential Scanning Calorimetry (DSC)

The thermal behaviour of POS-NP and BC-POS-NP were investigated to find a relationship between the thermal stability of bioactive compounds and the properties of pure polymer and BC-POS-NP. All analyses were carried out after the nanoparticles were freeze-dried.

**Figure 6:**
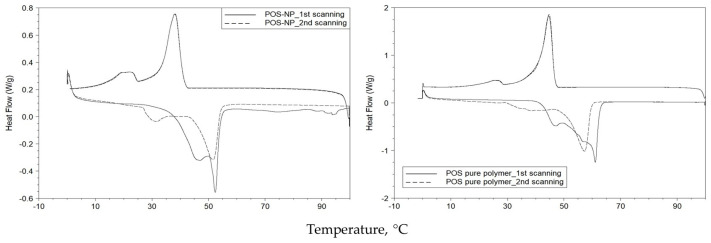
DSC thermogram of POS-NP and POS pure polymer; temperature ranges from 0 °C to 100 °C and from 100 °C to 0 °C.

All samples were scanned twice (from 0 to 100 °C and cooling again to 0 °C), and the differences between the thermograms obtained from the first and second scans were observed for POS pure polymer and POS-NP systems (Figure 6). The second run was used for DSC parameters calculations [55]. Tm of POS-NP for the second scan was approximately 1–2 °C lower than the first run. However, this difference was much higher for the POS pure polymer (approx 4–5 °C). The same occurs with the BC-POS-NP systems. Cho [62] reported the same phenomena for the retinol-encapsulated aliphatic polyester nanoparticles. Therefore, only the second run was used for DSC parameter calculations (old paper). 

The variation in the thermal behaviour of POS-NP and POS pure polymer was evaluated by changes in Tm, Tc, and polymer melting (ΔHm) and crystallization (ΔHc) enthalpies. The results showed that Tm and Tc of POS-NP decreased by approximately 11 and 18%, regarding the pure polymer. The melting enthalpies of POS-NP and POS pure polymer were 26.0 ± 1.78 Jg^−1^ and 63.4 ± 5.0 Jg^−1^, respectively, indicating nearly 58% energy reduction for the phase change of nanoparticles compared with the pure polymer. The same decrease was observed for crystallization enthalpies. In addition, ΔHc of POS-NP was 59% lower than for the pure polymer in its powder form. 

**Figure 7:**
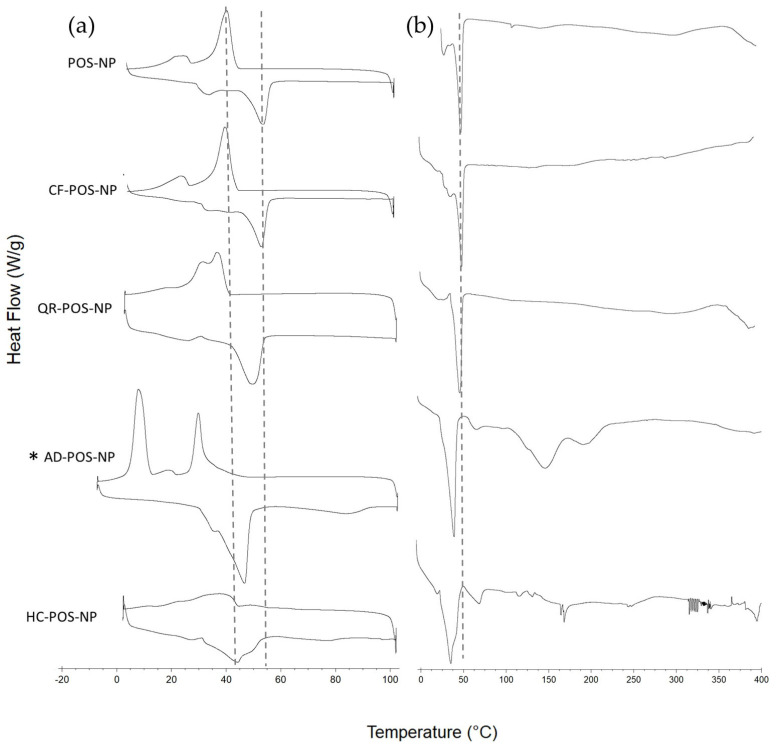
DSC thermogram for POS-NP and BC-POS-NPs: (**a**) from 0 °C to 100 °C and from 100 °C to 0 °C; (**b**) from 0 °C to 400 °C. * The temperature regime for AD-POS-NP (**a**) from −10 °C to 100 °C.

**Figure 8:**
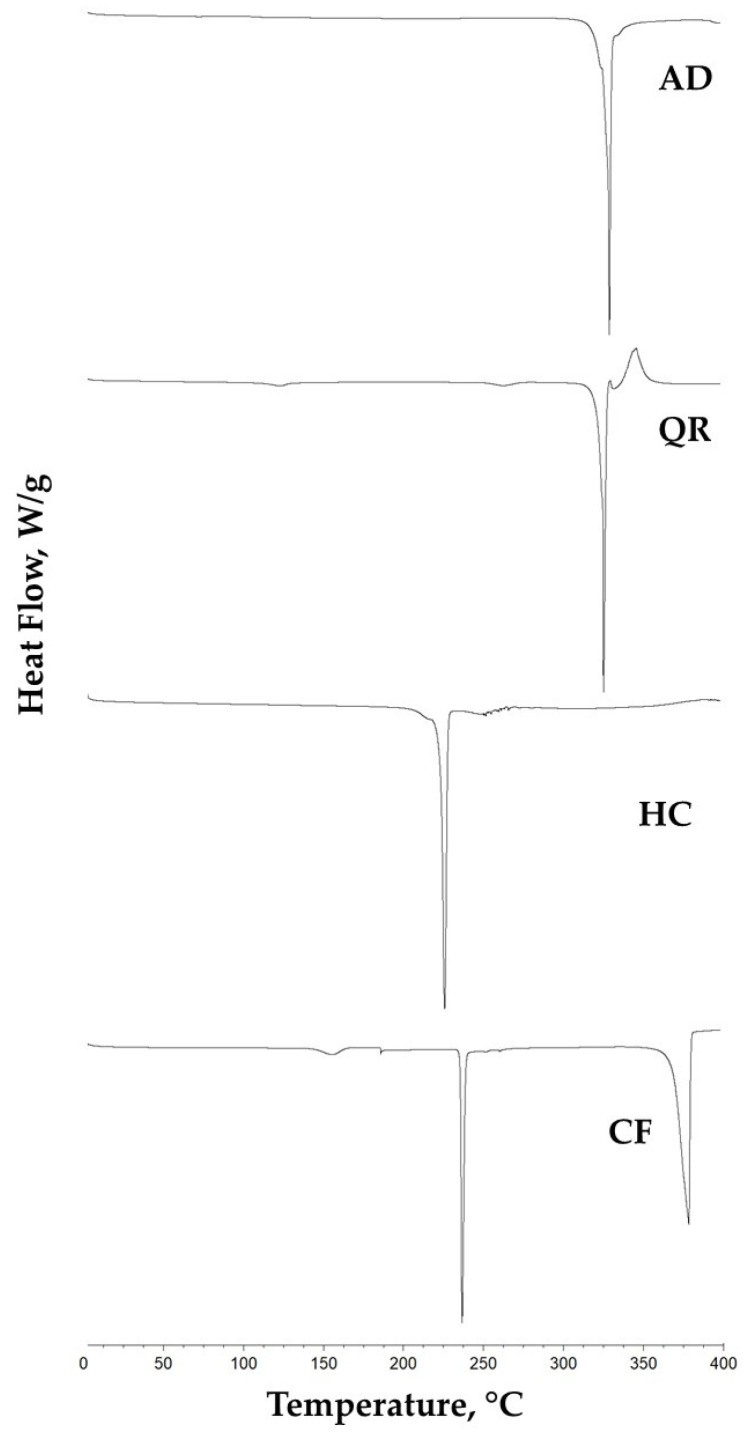
DSC thermogram for pure compounds.

DSC experiments were further conducted to obtain the thermograms of BC-POS-NPs (Figure 7a, Table 3) using two different temperature regimes, first heated and cooled in the range from 0 to 100 °C to characterise the crystalline and amorphous content of the NP matrix without NP degradation and afterwards additionally heated above the pure compounds’ melting points (until 400 °C, Figure 7b) to evaluate the BCs solubilisation into the NP matrix. As the highest Tm of pure BCs was observed for AD (Tm 328.4 °C, Figure 8), all samples were heated to 400 °C.

The nature of the bioactive compound influenced the depression of Tm and Tc for all BC-POS-NP, with an exception for CF-POS-NP (Table 3, Figure 7a). The CF-POS-NP (Figure 7a) has phase changes that are very similar to those of the no-loaded POS-NP (Figure 6), which was expected regarding the low encapsulation efficiency of this system (Table 2). QR-POS-NP showed a change in the broadness of melting and crystallization peaks. However, the DSC thermogram followed a pattern similar to POS-NP and CF-POS-NP for both temperature regimes. 

Tm of HC-POS-NP shift left at 9.62 °C from Tm of empty POS-NP, followed by the broadness of the melting peak, while the crystallization peak almost disappeared (Figure 7a). DSC scan of HC-POS-NP to 400 °C showed two peaks, the first at 42 ± 0.63 °C and the second at 73.94 ± 0.26 °C (Figure 7b). Multiple peaks were observed also for AD-POS-NP systems, at 44.06 ± 0.02 °C, 146.48 ± 2.00 °C, and 193.61 ± 6.36 °C (Figure 7b). Additionally, for AD-loaded particles, in the temperature range up to 100 °C, during the crystallization two peaks appeared at 26.66 ± 0.36 °C and 4.54 ± 0.45 °C (Figure 7a, Table 3). 

Multiple peaks in the DSC thermogram may suggest the polymorphic behaviour of these two systems. The structure of polymorphic substances affects medical and pharmaceutical properties such as bioavailability, suitability for required formulations, structural stability, and shelf-life [63]. Polymorphism has been determined for some polyesters having short polymethylene sequences (i.e., poly(ethylene succinate), PBS, and poly(butylene adipate)) [18].

Melting points were 224.6 °C, 235.9 °C, 325.2 °C, and 328.4 °C for pure HC, CF, QR, and AD, respectively (Figure 8). When the Tm peak of the pure compound (Figure 8) does not appear in the thermogram of the nanoformulation or the physical mixture, the drug is converted from crystalline to amorphous phase, confirming its complete solubilisation into nanoparticle formulations [49]. All observed system endothermic peaks of BC pure compounds disappear during the scan until 400 °C (Figure 7b). 

**Figure 9:**
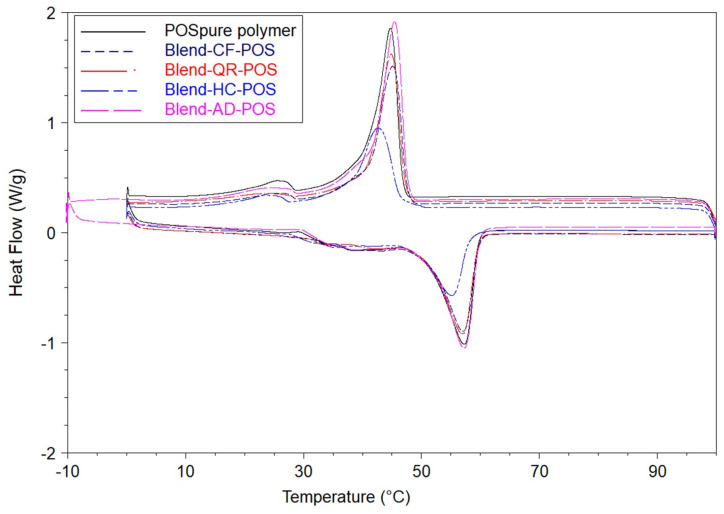
DSC thermogram for blends of POS pure polymer and blends of pure BCs (BC: CF, QR, HC, and AD) with POS pure polymer: temperature range from 0 °C to 100 °C and from 100 °C to 0 °C; BC concentration 0.5 wt%.

The DSC thermogram of the physical blends of pure polymer and pure bioactive compounds (Figure 9) showed only a slight Tm left shift of 2.18 °C for the HC–polymer mixture, while for the other nanoformulations, the change was less than 0.5 °C (Figure 9).

### 3.4. FTIR Analysis

**Figure 10:**
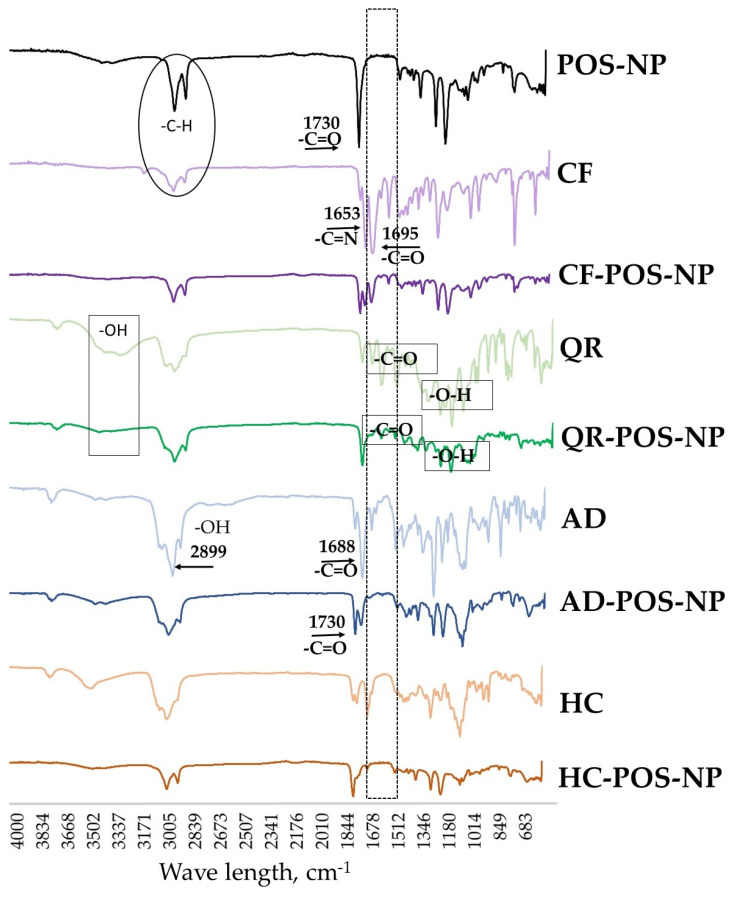
FTIR spectra of pure bioactive compounds (BC: CF, QR, AD, HC) and BC-POS-NPs.

FTIR spectroscopy was carried out on the pure BCs, BCs-loaded nanoparticles, and empty nanoparticles (POS-NP) to compare and evaluate structural properties (Figure 10). Regarding the FTIR spectra of the pure BCs and drug-loaded BC-POS-NP, changes in the concavity and intensity of the peaks denote the interaction of the drug with the polymers.

The FTIR spectrum of CF peaks at 3600 cm^−1^ due to N–H stretching vibration. Aromatic C–H stretch appears at 3113 cm^−1^ and 2953 cm−1. The peaks at 1695 cm^−1^ and 1651 cm^−1^ correspond to the C=O stretch in cyclic hydrocarbons and the C=N stretch in cyclic hydrocarbons, respectively. The region between 2800 cm^−1^ and 3000 cm^−1^ contains two peaks of 2854 and 2924 cm^−1^, of which the latter can be correlated with the asymmetric stretching of C–H bonds of methyl (–CH_3_) group in the caffeine molecule [64]. The FTIR spectrum of drug-loaded nanoparticles showed new peaks (1699 cm^−1^ and 1651 cm^−1^) where the C=O and C=N groups of cyclic hydrocarbons interacted with the polymer matrix and were not observed in POS-NP. 

FTIR spectra of QR-loaded POS-NP nanoparticles show quercetin-associated peaks [65], with –OH group stretching (3400–3500 cm^−1^), –C=C aromatic bending and stretching (1100–1600 cm^−1^), and –OH phenolic bending (1200–1400 cm^−1^) (Figure 10). These peaks are absent in POS-NP nanoparticles. 

Due to the conjugation of carboxylic acid to the aromatic ring, the C=O stretch of AD was at 1688 cm^−1^. The polymer and drug-loaded nanocarrier presented a −C=O ester stretch peak at 1730 cm^−1^. The strong peak at 2899 cm^−1^ was due to the AD carboxylic OH stretch. The peak at 2926 cm^−1^ was assigned to the polymer −C-H stretch. For the nanocarrier, the peaks at 2922 and 2978 cm^−1^ might originate from the drug OH and polymer CH vibration, respectively. Nevertheless, these peak shifts signified the potential hydrophobic and hydrogen bonding interactions between AD and POS-NP.

The FTIR spectrum of HC showed distinct peaks at 3410 cm^−1^, which confirms the presence of –OH groups; aliphatic C–H stretching was observed at 2970 cm^−1^ as well as 2908 cm^−1^, C=O was observed at 1705 cm^−1^ and 1640 cm^−1^, –CH_2_ bending was seen at 1454 cm^−1^, and, lastly, the spectrum showed C–O at 1047 cm^−1^ [66]. Lower peak intensity and slight shifting of the presence of –OH groups of HC were observed in HC-POS-NP.

### 3.5. ^1^H NMR Analysis of the BCs-POS-NPs Structure

**Figure 11:**
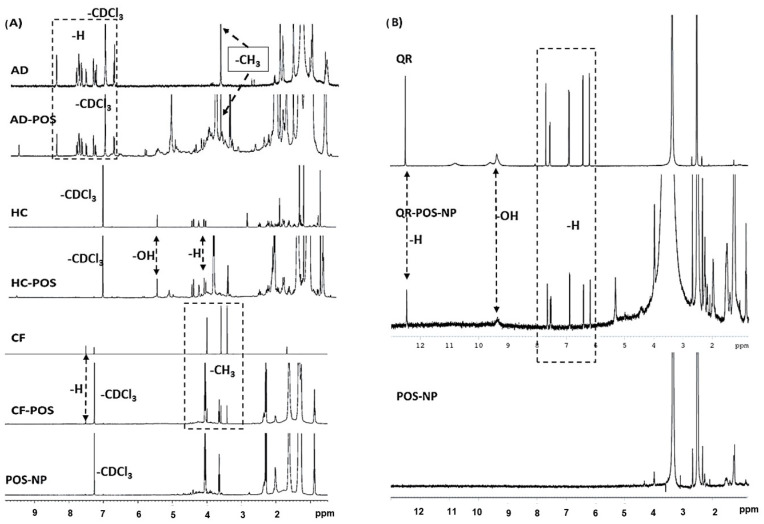
^1^H NMR spectra of BC-POS-NPs (BC: AD, HC, CF; CDCl3, 400 MHz, 25 °C) (**A**), and QR-POS-NP (DMSO-d6, 400 MHz, 25 °C) (**B**).

In the present study, ^1^H NMR spectroscopy was used to analyse the interaction between BCs and POS-NPs. Figure 11A,B presents a typical 400 MHz ^1^H NMR spectrum of BC-POS-NPs, with spectra obtained for BCs and POS-NP included for comparison. 

In the ^1^H NMR spectrum of POS-NP (CDCL3 solvent), it was possible to identify a diol methylene multiplet at 1.30–1.45 ppm, a diacid methylene multiplet at 1.55–1.70 ppm, and a methylene multiplet adjacent to the carbonyl group at 2.30–2.40 ppm. Additionally, a methylene triplet adjacent to the hydroxide group at 3.60–3.70 ppm corresponding to the C-linked Hs of the terminal hydroxyl of the polymer may also refer to the unreacted dialcohol or dialkyl terminal hydroxyl, and also a methylene triplet adjacent to the oxygen of the ester group at 4.00–4.20 ppm [23].

The ^1^H NMR spectrum of caffeine consists of the sole ring H signal (7.58 ppm) and the signals from three methyl groups (3.37, 3.55, and 4.01 ppm) [67]. The ^1^H-NMR spectrum of quercetin in DMSO-d6 exhibits H resonance peaks at 12.5, 7.70, 7.58, 6.91, 6.44, and 6.22 ppm (Figure 11A), which is attributed to the C-5 OH proton of flavonoids. The C-3, C-5 OH resonances appear in 9.18 ppm. Resonance peaks of H signals were detected for pure AD and AD-POS-NP in the range from 8.61 to 7.12 ppm, and also resonance for acetyl group at 3.87 ppm. 1HNMR spectra resonances peaks of H signals at 1.30, 1.95, and 4.08 ppm, as well as –OH resonance signal at 4.6 ppm and 5.46 ppm were detected in both HC and HC-POS-NP. However, the lack of the resonance for the H signal at 3.1 ppm was observed in HC-POS-NP. Resonance peaks at the same chemical shift, characteristic of free BCs, are also detected in the spectra of the BCs-POs-NP samples. This fact indicates that these resonances can be attributed to free BC molecules, encapsulated by the polymer, that retain enough mobility within the polymer matrix microenvironment so that their chemical shift is not disturbed when compared to the free molecules in solution [68].

For the AD-POS-NP, HC-POS-NP, and CF-POS-NP two resonance signals appear at 6.08 and 6.02 ppm, which can indicate drug interaction with the polymer. These signals were not observed with the QR-POS-NPs where DMSO-d6 was used as a solvent (Figure 11B). Future NMR spectroscopy data will be acquired to understand these interactions. 

However, the BC-POS-NPs spectrum obtained showed additional NMR peaks which indicated the presence of Span 80, a surfactant used in the NP production process. Since the samples were only centrifuged once, it is possible that some surfactant remains in the synthesised particles.

### 3.6. In Vitro Antioxidant Activity

**Figure 12:**
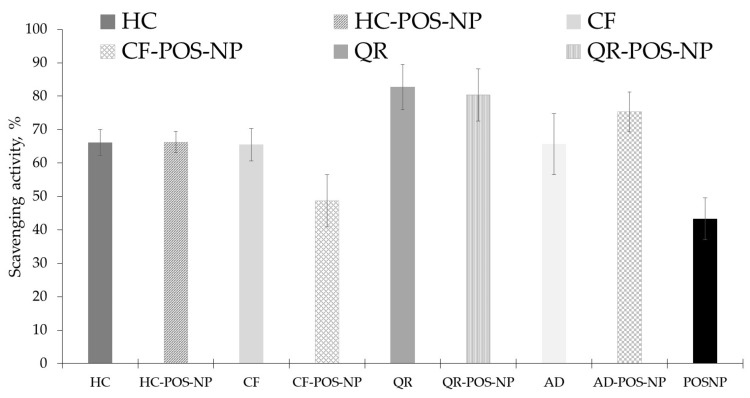
Antioxidant activity of pure BC used in the preparation of the polymeric nanoparticles BC-POS-NP (BC, 0.5 wt%), and of a pure BC solution (5 mg mL^−1^).

The DPPH assay was utilized to evaluate the scavenging activity (SA) of pure BCs and BC-POS-NPs, as illustrated in Figure 12. It is well known that pure BCs possess antioxidant properties, as reported by various studies [35,39,43,48]. For instance, caffeine has been shown to exhibit neuroprotective effects by reducing oxidative stress and apoptotic cell death in different models of neurodegeneration [39]. Additionally, it has been reported that QR can protect dermal cells from oxidative stress and free-radical-induced toxicity [35]. 

Figure 12 depicts the in vitro antioxidant activity of a pure BC solution, non-loaded POS-NPs, and BC-POS-NPs. The DPPH assay assesses the ability of DPPH radicals to scavenge oxygenated free radicals. The activity of free BCs was found to be 65.5 ± 4.90%, 66.1 ± 3.90%, 82.7 ± 6.72%, and 65.6 ± 9.2% for CF, HC, QR, and AD, respectively. It was observed that CF-POS-NP showed 26% lower SA compared to the pure compounds, but it was 11% higher than the SA of POS-NP (43.3 ± 6.3). Encapsulation in polymeric nanocarriers did not affect the SA of HC (<0.2%) and QR (<3%). For AD-PO-NP, antioxidant activity (75.3 ± 6.3) increased by 13% due to the drug encapsulation. The scavenging activities are statistically significant and ranged between 43.3 ± 6.3% and 82.7 ± 1.72% for POS-NP and QR pure, respectively (*p*-values of 0.131 and 0.370, respectively, with α = 0.05) 

Different behaviors for BCs may be attributed to the reactivity of antioxidants in dispersed systems, which depends on the location of radicals and antioxidants. It is important to note that antioxidants and radicals exhibit distinct solubility properties, which can have a significant impact on their reactions, especially in an unfavorable chemical environment.

Considering the primarily lipophilic nature of selected BCs, the more lipophilic the environment is for BCs, the better it is stabilized. Heins et al. claim that the position of phenolic antioxidants (e.g., QR) and radicals at interfaces determine their activity. They demonstrated that information on the solubilisation sites of antioxidants and radicals is essential to control the antioxidant activity at interfaces of drug delivery systems in cosmetics, pharmaceuticals (emulsions and carrier systems), and biological membranes [69].

### 3.7. Biocompatibility Studies

**Figure 13:**
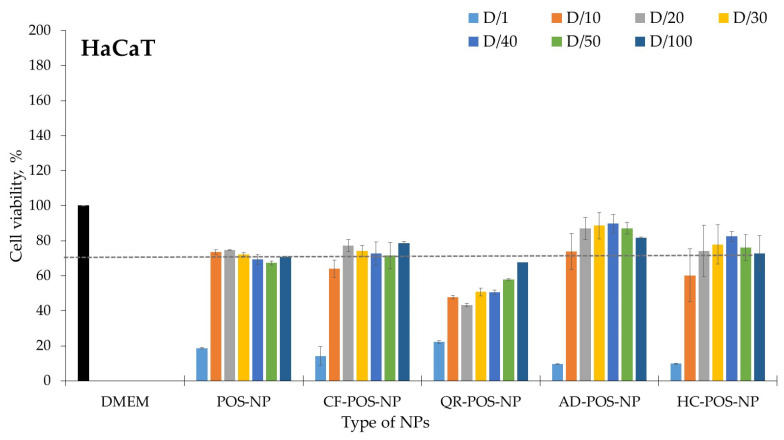
Cytocompatibility (viability %) of keratinocytes (HaCaT) for POS-NP, CF-POS-NP, QR-POS-NP, AD-POS-NP, and HC-POS-NP; D-dilution factor; D-NPs dilution; D/1, D/10, D/20, D/30, D/40, D/50 correspond to NP concentrations 25, 2.5, 1.25, 0.83, 0.63, 0.50, and 0.25 mg/mL^−1^ expressed by amount of total polymer. The dashed line presents the 70% cell viability limit for cell cytotoxicity (ISO 10993-5:2009 [60]).

**Figure 14:**
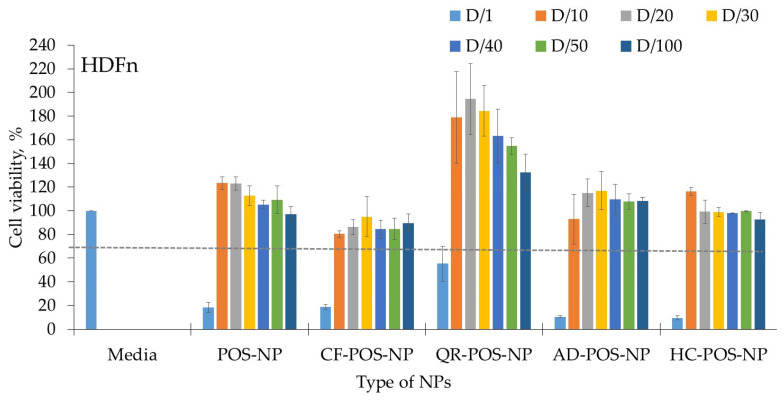
Cytocompatibility (viability, %) of fibroblasts (HDFn) for POS-NP, CF-POS-NP, QR-POS-NP, AD-POS-NP, and HC-POS-NP; D-dilution factor; D-NPs dilution; D/1, D/10, D/20, D/30, D/40, D/50 correspond NPs concentration 25, 2.5, 1.25, 0.83, 0.63, 0.50, and 0.25 mg/mL^−1^ expressed by amount of total polymer. The dashed line presents the 70% cell viability limit for cell cytotoxicity (ISO 10993-5:2009 [60]).

Determination of cell viability was used to test the biocompatibility of polymeric nanocarriers. BC-POS-NPs were evaluated against two main dermal lines, keratinocytes (HaCaT) and fibroblasts (HDFn). Several pharmacology studies have used these two cell lineages in well-defined experimental models [70]. The obtained results are illustrated in Figure 13 and Figure 14, respectively. Table 4 presents the nontoxic POS-NP and BC-POS-NP concentrations expressed by total polymer concentration in particles (mg mL^−1^). The polymer concentration in nanoemulsion was 25 mg mL^−1^ (2.5 wt%). For all systems, this concentration expressed toxicity for both cell lines (Figure 13 and Figure 14). Concentration-dependent cytotoxicity was observed only for QR-POS-NP formulations. It has been shown that QR has potentially toxic effects, including its mutagenicity, pro-oxidant activity, and mitochondrial toxicity [71].

As shown in Figure 13, QR-POS-NP nanoparticles showed cytotoxicity for all explored nanoparticle concentrations towards HaCaT cells. POS-NP particles did not have any cytotoxicity at high concentrations (2.5 mg mL^−1^) regarding both cell lines, which was expected as the biocompatibility of the pure polymer showed the same high biocompatibility. Slightly higher toxicity towards HaCat cells was shown by CF-POS-NP and HC-POS-NP. It was verified that the cell viability results for NLC formulations prepared with each POS-NP and BC-POS-NP were all statistically significant with *p*-values ≥ 0.05, with one exception for the QR-POS-NPs (333 ± 34 nm, *p*-value = 0.020, α = 0.05). For example, HC-loaded Dextran-PLGA micelle had no cytotoxicity on fibroblasts cells, even at high concentrations of 200 μg mL^−1^ [46]. Drug delivery systems usually reduce the toxic effects on healthy cells.

After exposition to the same concentration of POS-NP and BC-POS-NP, the metabolic activity of HDFns increase (Figure 14). It was verified that the cell viability results for nanoparticle formulations prepared with each POS-NP and BC-POS-NP were all statistically significant, with *p*-values ≥ 0.05, with one exception for the QR-POS-NP cytotoxicity effect on HDFn. The HDFn (fibroblasts) cell viability exposed to QR-POS-NPs were 55.3 ± 14.9%, 178.7 ± 38.7%, 194.9 ± 29.9%, 184.3 ± 21.5%, 163.2 ± 22.6%, 154.4 ± 7.3%, and 132.3 ± 15.5%, with *p*-values of 0.0020, 0.0241, 0.0167, 0.0121, 0.0089, 0.0020, and 0.0038 for concentrations 25, 2.5, 0.83, 0.63, 0.50, and 0.25 mg mL^−1^ expressed by amount of total polymer. This can be attributed to the heterogeneity of QR-POS-NP systems (high PDI and tendency for particle agglomeration previously described in Section 3.2.1 and Section 3.2.2), which leads to variable interaction with the cells. However, this impact was not observed for the HaCat cells in the same system.

The lower value of cell viability of HaCaT cells compared to HDFn cells was also previously observed by authors for lipid particles [51,52]. The cell viability for pure compounds in quantities and ratios as in the final particle formulations (BC-POS-NP) was less than 40%. Therefore, encapsulation in POS-NPs decreases cell cytotoxicity of the explored BCs.

### 3.8. In Vitro Permeability Studies of BC-POS on RHE

An in vitro skin permeation study using reconstructed human epidermis (RHE) was performed to investigate the effect of the incorporation of selected BCs in POS-NP on upper skin layer (epidermis) using the Franz diffusion cell over 24 h. The in vitro experiments were performed according to the OECD Test Guideline 428 [58].

The permeation profiles of BC-POS-NPs were studied to evaluate the release profile and skin absorption properties of four selected bioactive compounds using the RHE model that may replicate the topical absorption by native human epidermis. This study was performed in sink conditions, using a concentration of 0.5% of each active substance in the same POS-NP composition (Table 1).

**Figure 15:**
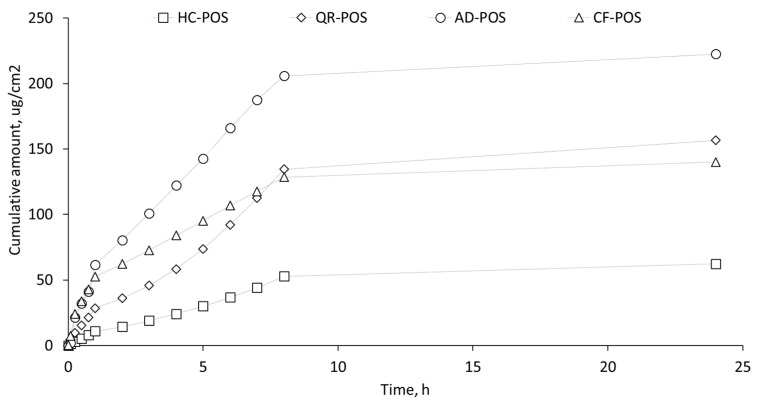
In vitro permeation profiles of BC-POS-NP. Cumulative amounts released from NPs in an RHE model. Plots were obtained from five experiments with each tested compound running under the same experimental conditions with three replicates. Error bars represent the mean ± SD value.

The release profiles of AD, QR, HC, and CF were plotted to compare the absorption of each BC-POS-NP formulation (Figure 15). Additionally, the permeation parameters, including the permeation rate at steady state (J, μgcm^−2^ h−^1^) and the skin permeability coefficient (P, ×10^−4^ cm h^−1^), were determined and listed in Table 5.

All BCs permeated through the RHE model, demonstrating a controlled release over 24 h. The kinetics of BC-POS-NP release from the NP matrix were biphasic (Figure 15). An initial small burst (till 1 h) followed by sustained release of the drug was observed. This phenomenon might be due to the rapid release of BCs attached at the surface of NPs at the initial hour, followed by drug diffusion from water-filled pores and polymeric matrix, as well as drug release attributed to the degradation of the matrix [22,72,73]. Guo et al. [62] reported the same biphasic release profile for AD encapsulated in acid-responsive polymer (Eudragit^®^ EPO) nanocarriers on silicone membrane and full-thickness pig skin.

We found that at normal physiological conditions (pH 7.4 and 37 °C temperature), the maximum cumulative amount of 92.47%, 85.92%, 84.81%, and 91.65% was released in 8 h with initial burst release of 27.76%, 18.24%, 17.58%, and 31.57% for AD, QR, HC, and CF, respectively. Permeation parameters (Table 5) show increasing permeability of AD compared with QR, HC, and CF. Baksi et al. [73] found that at normal physiological conditions, a maximum cumulative amount of 67.28% QR encapsulated in chitosan nanoparticles was released in 12 h with an initial burst release of 29.68% of QR in 1 h. The permeation rate of CF was surprisingly higher than QR and HC (Figure 15, Table 5), which are more lipophilic and more integrated in POS-NP (Table 5).

CF is correctly characterised as an amphiphilic molecule (logP = −0.07), which, due to specific lipophilic moieties, is able to partition into the lipid bilayer and diffuse across into the cell [74]. AD is highly lipophilic with a LogP 8 [75], while HC and QR have LogP 1.61 and 1.59, respectively [76,77]. LogP is a crucial factor governing passive membrane partitioning; an increase in LogP enhances permeability.

The active substance should be carried through the SC and accumulate there. A concentration gradient favours this permeation and allows for rapid diffusion and a higher flux [78]. The diffusion process depends on the physicochemical properties of the BCs, primarily on their molar mass and partition coefficient, and then their solubility in the aqueous receptor fluid. For cosmetic purposes, NPs should not permeate the epidermis as stated in Regulation No. 1223/2009 of the European Parliament and the Council [60]. Instead, as nanocarriers, they should enhance the permeation of active compounds.

**Figure 16:**
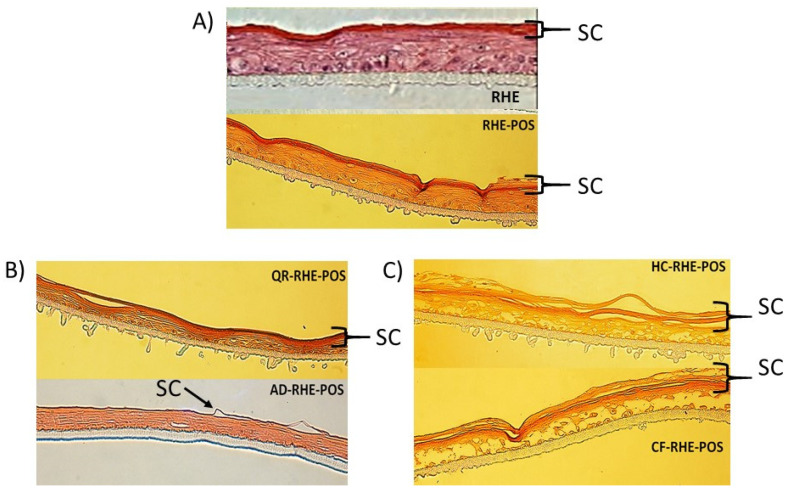
Comparative histological aspect of BC-POS-NP permeation on RHE. RHE—reconstructed human epidermis; RHE-POS—reconstructed human epidermis with POS-NP (**A**), QR-RHE-POS and AD-RHE-POS—reconstructed human epidermis with AD and QR loaded POS-NPs (**B**), HC-RHE-POS and CF-RHE-POS—reconstructed human epidermis with HC and CF loaded POS-NPs (**C**); SC—stratum corneum; hematoxylin–eosin staining; Nikon Eclipse TE2000-S bright field microscope (Nikon instruments, Melville, NY, USA) magnification 20×.

RHE was histologically examined after 24 h to evaluate the interaction of proposed BCs-POS-NPs on the skin barrier (Figure 16). The purpose of this study was to investigate the comparative effect of POS-NPs and BC-POS-NPs on stratum corneum, which directly links to drug penetration [78]. The stratum corneum (SC, Figure 16) represents the top layer of the skin with “brick” (corneocytes) and mortar (lipid matrix) structure, and is generally assumed to be the main barrier for NP absorption. The interaction with the SC may change three-dimensional conformation of polymeric NPs leading to adsorption, destabilization, or agglomeration. The tendency of QR-POS-NPs to form agglomerates could influence their lower permeation in comparison with other systems. The hydrophilicity of the drug delivery system is crucial for interaction with SC.

The morphology of RHE did not change by the permeation of unloaded POS-NPs (Figure 16A). The slight increase in thickness of the stratum corneum due to the hydration effect was the consequence of the NPs covering the surface of the skin, which reduces the evaporation of water from the skin, thus increasing the moisture content of the skin. This effect was also observed for QR-POS-NP compared with the untreated skin where the tissues presented also a uniformly layered SC with some interlamellar gaps between the SC and the lower layer of the epidermis (Figure 16B).

After the application of the selected systems, the thickness and morphology of SC significantly change after 24 for HC-POS-NPs and CF-POS-NPs. Lower layers of the epidermis were disintegrated, indicating these systems’ toxic effect on RHE after 24 h. While cytotoxicity was not observed after 3 h for either of the two systems investigated for 2D dermal cell assays (Figure 13 and Figure 14), a more extended exposition of these systems on the skin has a toxic effect. It could explain the faster permeation rate for CF-POS-NP in comparison with the QR-POS-NP (Figure 15).

When AD-POS-NP was applied, we observed a significant decrease in the thickness of the SC, while the lower layers of the epidermis remained unchanged. This suggests that the higher permeation rate of AD (Figure 15) may be due to the reduced thickness of the SC, a direct result of the application of AD-POS-NP.

Based on permeation and histology studies, HC-POS-NPs and CF-POS-NPs were shown to not be suitable for dermal applications with the explored drug concentration. To better understand the administration and absorption of AD-POS-NPs and QR-POS-NPs on RHE, fluorescent labelling of the particles will be performed.

## 4. Conclusions

Poly(octamethylene suberate) (POS) nanoparticles (POS-NPs) were successfully synthesised using the oil-in-water one-step miniemulsion process without using organic solvents to encapsulate bioactive compounds. Encapsulation of hydrophilic (CF) and three hydrophobic compounds (QR, HC, AD) showed the apparent affinity of POS-NPs to encapsulate hydrophobic compounds. However, the chemical structure of hydrophobic compounds additionally influenced the nanoparticle’s physicochemical characteristics and entrapment efficiency. Regarding physicochemical characteristics (mean size, particle charge, and PDI), nanoparticles loaded with HC and AD showed better stability within experimental conditions. QR formed heterogeneous microparticle emulsions with very high PDI, indicating possible agglomerate formation, possibly due to the multiple presence of –OH groups in its structure. The authors did not observe the presence of agglomerate in QR-encapsulated lipid particles using the same methodology and QR concentration [48].

DSC, NMR, and FTIR confirmed drug incorporation in the POS-NP matrix. The degree of crystallinity moved to a more amorphous structure and polymorphism (Ad-POS-NP). However, the DSC thermogram of CF-POS-NP was more similar to the POS-NP, probably due to this system’s low encapsulation efficiency.

The antioxidant activity of BC-POS-NP was increased compared to that of the free bioactive compound. Drug release from the POS-NPs matrix shows a biphasic profile on RHE, with fast release in the first hour followed by controlled diffusion.

Biocompatibility on 2D dermal cells and 3D safety and permeation studies showed the impact of the BC-POS-NP concentration on cytotoxicity. Encapsulation of BCs decreases dermal cell cytotoxicity for all systems except for QR-POS-NPs and keratinocytes (HaCaT). Only the cytotoxicity of the QR-POS-NP on HaCaT cells increased compared to that observed for POS-NP systems, which may be due to higher QR adsorption on NP surfaces and contact with keratinocyte cells. Permeation profiles on the 3D RHE model showed no toxic effect of no diluted POS-NPs, QR-POS-NPs, and AD-POS-NPs. The stratum corneum, an outmost layer of the epidermis, is the principal skin barrier and protection from environmental (e.g., UV radiation, microbes) impact, and it was expected to have a less toxic effect on 3D RHE than on dermal cells. However, skin tissue damage was observed for HC-POS-NPs and CF-POS-NPs, mostly in lower levels of the RHE. The permeation test was done during 24 h, and time-dependent exposure with different BC-POS-NP concentrations will be done to optimise loaded drug dosage.

Summary: We proved that POS-NPs may be a promising drug delivery system for topical applications. The current studies involve optimising BC concentrations regarding the physicochemical characteristics and biocompatibility studies on 3D models using experiment design.

## Figures and Tables

**Table 1:** Composition of nanoparticle formulations.

Formulation	Polymer, wt%	Surfactant, wt%	Bioactive Compound, wt%	Water, wt%
POS-NP	2.5	2	-	95.5
BC-POS-NP	2.5	2	0.5	95

**Table 2:** Encapsulation efficiency (EE, %) and drug loading (DL, %) for BC-POS-NPs with 0.5 wt% of bioactive compounds.

BC-POS-NP	EE, %	DL, %
CF-POS-NP	28.30 ± 1.81	36.11 ± 1.48
HC-POS-NP	83.09 ± 4.32	62.42 ± 1.29
QR-POS-NP	99.90 ± 0.10	66.65 ± 3.56
AD-POS-NP	99.95 ± 0.04	66.66 ± 4.87

**Table 3:** Melting (T_m,_ °C), crystallization (T_c,_ °C) peaks temperatures, glass transition of melting (T_gm_, °C), glass transition of crystallization (T_gm_, °C) temperatures, and melting (ΔH_m_, J/g) and crystallization (ΔH_c_, J/g) enthalpies of free (POS-NP) and BC-POS-NPs.

BC-POS-NP	T_gm_ (°C)	T_m_ (°C)	ΔH_m_ (J/g)	Tc (°C)	ΔH_c_ (J/g)	T_gc_ (°C)
CF	30.39 ± 3.44	51.09 ± 0.12	21.20 ± 2.01	37.62 ± 0.05	34.46 ± 2.50	20.04 ± 0.05
QR	23.91 ± 4.98	47.07 ± 0.27	35.10 ± 4.58	30.13 ± 3.99	33.65 ± 0.27	12.17 ± 0.05
AD	30.64 ± 0.58	43.96 ± 0.11	46.63 ± 1.68	26.66 ± 0.36 4.54 ± 0.45	24.28 ± 7.78 42.97 ± 2.95	17.95 ± 0.02
HC	22.52 ± 0.03	41.48 ± 0.57	31.16 ± 3.80	32.66 ± 2.62	16.23 ± 2.61	17.44 ± 1.05
POS	31.85 ± 3.44	51.1 ± 0.39	26.0 ± 1.78	36.3 ± 1.25	36.64 ± 2.72	18.17 ± 2.96

**Table 4:** Nontoxic total POS-NP and BC-POS-NP concentration (mg/mL^−1^) for HaCaT and HFDn cell lines expressed by amount of total polymer (mg/mL^−1^).

Total Polymer, mg/mL^−1^	HaCaT	HDFn
POS-NP	≤2.5	≤2.5
CF-POS-NP	≤1.25	≤2.5
HC-POS-NP	≤1.25	≤2.5
QR-POS-NP	toxic	≤2.5
AD-POS-NP	≤2.5	≤2.5

**Table 5:** Cumulative percentage amount and permeability parameters (permeation rate at steady state, J (µgcm^−2^ h^−1^) and skin permeability coefficient, P (×10^−4^, cmh^−1^) for BCs released from BC-POS-NPs through an in vitro RHE model. The initial concentration of BCs in Franz cell donor system was 5 mg mL^−1^.

	AD-POS-NP	CF-POS-NP	QR-POS-NP	HC-POS-NP
Cumulative amount (µgcm^−2^), 24 h	439.2 ± 108.2	140.1 ± 4.14	114.73 ± 20.2	62.38± 3.67
J (µgcm^−2^ h^−1^)	∗118.0 ± 69.4 ^∙^43.8 ± 3.90	∗50.44 ± 7.21 ^∙^11.12 ± 1.51	∗23.18 ± 5.24 ^∙^16.52 ± 2.87	∗10.54 ± 2.15 ^∙^6.36 ± 0.483
P (×10^−4^, cm h^−1^)	∗235.8 ± 126.7 ^∙^42.38 ± 7.81	∗100.88 ± 15.6 ^∙^2.224 ± 0.568	∗46.36 ± 2.56 ^∙^33.04 ±5.21	∗21.08 ± 4.30 ^∙^6.36 ± 0.966

Note: ∗ from 2 h to 8 h; ∙ from 0 to 1 h; R^2^ for all curves was above 98%.

## Data Availability

The data presented in this study are available on request from the corresponding author. The data are not publicly available due to [a new paper preparation].

## References

[1] Yang R., Wei T., Goldberg H., Wang W., Cullion K., Kohane D.S. (2017). Getting Drugs Across Biological Barriers. Adv. Mater..

[2] Narasimha Murthy S., Shivakumar H.N. (2010). Topical and Transdermal Drug Delivery. Handbook of Non-Invasive Drug Delivery Systems.

[3] Vogt A., Wischke C., Neffe A.T., Ma N., Alexiev U., Lendlein A. (2016). Nanocarriers for drug delivery into and through the skin—Do existing technologies match clinical challenges?. J. Control. Release.

[4] Raszewska-Famielec M., Flieger J. (2022). Nanoparticles for Topical Application in the Treatment of Skin Dysfunctions-An Overview of Dermo-Cosmetic and Dermatological Products. Int. J. Mol. Sci..

[5] Kumari A., Yadav S.K., Yadav S.C. (2010). Biodegradable polymeric nanoparticles based drug delivery systems. Colloids Surf. B Biointerfaces.

[6] Elsabahy M., Wooley K.L. (2012). Design of polymeric nanoparticles for biomedical delivery applications. Chem. Soc. Rev..

[7] Ul-Islam M., Khan S., Ullah M.W., Park J.K., Thakur V.K., Thakur M.K. (2015). Structure, Chemistry and Pharmaceutical Applications of Biodegradable Polymers. Handbook of Polymers for Pharmaceutical Technologies.

[8] Mota A.H., Rijo P., Molpeceres J., Reis C.P. (2017). Broad overview of engineering of functional nanosystems for skin delivery. Int. J. Pharm..

[9] Gupta N., Gupta G.D., Singh D. (2022). Localized topical drug delivery systems for skin cancer: Current approaches and future prospects. Front. Nanotechnol..

[10] Seyednejad H., Ghassemi A.H., van Nostrum C.F., Vermonden T., Hennink W.E. (2011). Functional aliphatic polyesters for biomedical and pharmaceutical applications. J. Control. Release.

[11] Silvers A.L., Chang C.C., Emrick T. (2012). Functional aliphatic polyesters and nanoparticles prepared by organocatalysis and orthogonal grafting chemistry. J. Polym. Sci. Part A Polym. Chem..

[12] Karavelidis V., Karavas E., Giliopoulos D., Papadimitriou S., Bikiaris D. (2011). Evaluating the effects of crystallinity in new biocompatible polyester nanocarriers on drug release behavior. Int. J. Nanomed..

[13] Rawat M., Singh D., Saraf S. (2006). Nanotechcarriers: Promising vehicle for bioactive drugs. Biol. Pharm. Bull..

[14] Hickey J.W., Santos J.L., Williford J.-M., Mao H.-Q. (2015). Control of polymeric nanoparticle size to improve therapeutic delivery. J. Control. Release.

[15] Fattal E., Vauthier C., Swarbrick J. (2006). Drug Delivery: Nanoparticles in Encyclopedia of Pharmaceutical Technology.

[16] Kamaly N., Yameen B., Wu J., Farokhzad O.C. (2016). Degradable controlled-release polymers and polymeric nanoparticles: Mechanisms of controlling drug release. Chem. Rev..

[17] Díaz A., Katsarava R., Puiggalí J. (2014). Synthesis, properties and applications of biodegradable polymers derived from diols and dicarboxylic acids: From polyesters to poly(ester amide)s. Int. J. Mol. Sci..

[18] Gestí S., Casas M.T., Puiggali J. (2008). Single crystal morphology and structural data of a series of polyesters derived from 1,8-octanediol. Eur. Polym. J..

[19] Washington K.E., Kularatne R.N., Karmegam V., Biewer M.C., Stefan M.C. (2017). Recent advances in aliphatic polyesters for drug delivery applications. Wiley Interdiscip. Rev. Nanomed. Nanobiotechnol..

[20] Zhang Z., Tsai P.C., Ramezanli T., Michniak-Kohn B.B. (2013). Polymeric nanoparticles-based topical delivery systems for the treatment of dermatological diseases. Wiley Interdiscip. Rev. Nanomed. Nanobiotechnol..

[21] Guo C., Khengar R.H., Sun M., Wang Z., Fan A., Zhao Y. (2014). Acid-responsive polymeric nanocarriers for topical adapalene delivery. Pharm. Res..

[22] Pfluck A.C.D., de Barros D.P.C., Fonseca L.P. (2021). Biodegradable Polyester Synthesis in Renewed Aqueous Polycondensation Media: The Core of the New Greener Polymer-5B Technology. Processes.

[23] Fonseca L.J.P., Pfluck A.C.D., de Barros D.P.C. (2021). Synthesis of Polyesters in Aqueous Polymerization Media “from de Solid to Solid” via Biocatalysis. PCT/PT2020/050051 Published as WO 2021/137711 A1. https://patentscope.wipo.int/search/pt/detail.jsf?docId=WO21137711.

[24] Pfluck A.C.D., de Barros D.P.C., Oliva A., Fonseca L.P. (2022). Enzymatic Poly (octamethylene suberate) Synthesis by a Two-Step Polymerization Method Based on the New Greener Polymer-5B Technology. Processes.

[25] Casas M.T., Puiggalí J. (2009). Enzymatic degradation of poly(octamethylene suberate) lamellar crystals. Polym. Degrad. Stab..

[26] Shang Y., Li X., Jiang Z., Qiu Z. (2020). Synthesis, crystallization behavior and mechanical properties of novel biobased Poly(octamethylene succinate. Polym. Degrad. Stab..

[27] Rao J.P., Geckeler K.E. (2011). Polymer Nanoparticles: Preparation Techniques and Size-Control Parameters. Prog. Polym. Sci..

[28] Kang W., Choi D., Park T. (2019). Dietary Suberic Acid Protects Against UVB-Induced Skin Photoaging in Hairless Mice. Nutrients.

[29] Junnuthula V., Kolimi P., Nyavanandi D., Sampathi S., Vora L.K., Dyawanapelly S. (2022). Polymeric Micelles for Breast Cancer Therapy: Recent Updates, Clinical Translation and Regulatory Considerations. Pharmaceutics.

[30] Woodfield T.B., Malda J., de Wijn J., Péters F., Riesle J., van Blitterswijk C.A. (2004). Design of porous scaffolds for cartilage tissue engineering using a three-dimensional fiber-deposition technique. Biomaterials.

[31] Lu W., Kelly A.L., Miao S. (2016). Emulsion-based encapsulation and delivery systems for polyphenols. Trends Food Sci. Technol..

[32] Landfester K., Weiss C.K., Caruso F. (2010). Encapsulation by Miniemulsion Polymerization. In Modern Techniques for Nano- and Microreactors/-reactions. Advances in Polymer Science.

[33] Magar R.T., Sohng J.K. (2020). A Review on Structure, Modifications and Structure-Activity Relation of Quercetin and Its Derivatives. J. Microbiol. Biotechnol..

[34] Yang D., Wang T., Long M., Li P. (2020). Quercetin: Its Main Pharmacological Activity and Potential Application in Clinical Medicine. Oxidative Med. Cell. Longev..

[35] Wang W., Sun C., Mao L., Ma P., Liu F., Yang J., Gao Y. (2016). The biological activities, chemical stability, metabolism and delivery systems of quercetin: A review. Trends Food Sci. Technol..

[36] Kleemann R., Verschuren L., Morrison M., Zadelaar S., van Erk M.J., Wielinga P.Y., Kooistra T. (2011). Anti-inflammatory, antiproliferative and anti-atherosclerotic effects of quercetin in human in vitro and in vivo models. Atherosclerosis.

[37] Mukhopadhyay P., Prajapati A.K. (2015). Quercetin in anti-diabetic research and strategies for improved quercetin bioavailability using polymer-based carriers—A review. RSC Adv..

[38] Luo L., Lane M.E. (2015). Topical and transdermal delivery of caffeine. Int. J. Pharm..

[39] Simsolo E.E., Eroğlu İ., Tanrıverdi S.T., Özer Ö. (2018). Formulation and Evaluation of Organogels Containing Hyaluronan Microparticles for Topical Delivery of Caffeine. AAPS PharmSciTech.

[40] Milkova V., Goycoolea F.M. (2020). Encapsulation of caffeine in polysaccharides oil-core nanocapsules. Colloid. Polym. Sci..

[41] Makky A.M., El-Leithy E.S., Hussein D.G., Khattab A. (2022). Skin Targeting of an Optimized Caffeine Nanostructured Lipid Carrier with Improved Efficiency Against Chemotherapy Induced Alopecia. Int. J. App Pharm..

[42] Mehta A.B., Nadkarni N.J., Patil S.P., Godse K.V., Gautam M., Agarwal S. (2016). Topical corticosteroids in dermatology. Indian J. Dermatol. Venereol. Leprol..

[43] Kondiah P.P.D., Rants’o T.A., Mdanda S., Mohlomi L.M., Choonara Y.E. (2022). A Poly (Caprolactone)-Cellulose Nanocomposite Hydrogel for Transdermal Delivery of Hydrocortisone in Treating Psoriasis Vulgaris. Polymers.

[44] Rosado C., Silva C., Reis C.P. (2013). Hydrocortisone-loaded poly(ε-caprolactone) nanoparticles for atopic dermatitis treatment. Pharm. Dev. Technol..

[45] Malekhosseini S., Rezaie A., Khaledian S., Abdoli M., Zangeneh M.M., Hosseini A., Behbood L. (2020). Fabrication and characterization of hydrocortisone loaded Dextran-Poly Lactic-co-Glycolic acid micelle. Heliyon.

[46] Khalil S., Bardawil T., Stephan C., Darwiche N., Abbas O., Kibbi A.G., Nemer G., Kurban M. (2017). Retinoids: A Journey from the Molecular Structures and Mechanisms of Action to Clinical Uses in Dermatology and Adverse Effects. J. Dermatol. Treat..

[47] Ramezanhi T., Zhang Z., Michniak-Kohn B.B. (2017). Development and characterization of polymeric nanoparticle-based formulation of adapalene for topical acne therapy. Nanomed. Nanotechnol. Biol. Med..

[48] Jain A.K., Mehta P. (2023). Fabrication and functional attributes of nanotherapeutically engineered system for co-administration of adapalene and lycopene for enhanced acne management: An in vitro and in vivo investigation. Beni-Suef Univ. J. Basic Appl. Sci..

[49] Dreher F., Maibach H. (2001). Protective effects of topical antioxidants in humans. Curr. Probl. Dermatol..

[50] de Barros D.P.C., Reed P., Alves M., Santos R., Oliva A. (2021). Biocompatibility and Antimicrobial Activity of Nanostructured Lipid Carriers for Topical Applications Are Affected by Type of Oils Used in Their Composition. Pharmaceutics.

[51] de Barros D.P.C., Santos R., Reed P., Fonseca L.P., Oliva A. (2022). Design of Quercetin-Loaded Natural Oil-Based Nanostructured Lipid Carriers for the Treatment of Bacterial Skin Infections. Molecules.

[52] Adi-Dako O., Oppong Bekoe S., Ofori-Kwakye K., Appiah E., Peprah P. (2017). Novel HPLC Analysis of Hydrocortisone in Conventional and Controlled-Release Pharmaceutical Preparations. J. Pharm..

[53] Pinto F., de Barros D.P.C., Reis C., Fonseca L.P. (2019). Optimization of nanostructured lipid carriers loaded with retinoids by central composite design. J. Mol. Liq..

[54] Aydın A.A., Toprakçı G. (2021). Synthesis and characterization of new organic phase change materials (PCMs): Diesters of suberic acid. Sol. Energy Mater. Sol. Cells.

[55] Pinto F., Fonseca L.P., Souza S., Oliva A., de Barros D.P.C. (2020). Topical distribution and efficiency of nanostructured lipid carriers on a 3D reconstructed human epidermis model. J. Drug Deliv. Sci. Technol..

[56] Zoio P., Ventura S., Leite M., Oliva A. (2021). Pigmented Full-Thickness Human Skin Model Based on a Fibroblast-Derived Matrix forLong-Term Studies. Tissue Eng. Part C Methods.

[57] OECD (Organisation for Economic Co-operation and Development) (2004). Guidelines for Testing Chemicals-428 Skin Absorption: In Vitro Method.

[58] Shah V.P., Elkins J., Hanus J., Noorizadeh C., Skelly J.P. (1991). In vitro release of hydrocortisone from topical preparations and automated procedure. Pharm. Res..

[59] E.P.a.o.t. Council (2009). Regulation (EC) No 1223/2009 of the European Parliament and of the Council of 30 November 2009 on Cosmetic Products. Off. J. Eur. Union.

[60] ISO ISO 10993-5:2009: Biological Evaluation of Medical Devices—Part 5: Tests for In Vitro Cytotoxicity. https://www.iso.org/standard/36406.html.

[61] Choudhary A., Kant V., Jangir B.L., Joshi V.G. (2020). Quercetin loaded chitosan tripolyphosphate nanoparticles accelerated cutaneous wound healing in Wistar rats. Eur. J. Pharmacol..

[62] Cho E.C. (2012). Effect of polymer characteristics on the thermal stability of retinol encapsulated in aliphatic polyester nanoparticles. Bull. Korean Chem. Soc..

[63] Kumar A., Chauhan J., Dubey K.D., Sen S., Munshi P. (2022). Tuning Potency of Bioactive Molecules via Polymorphic Modifications: A Case Study. Mol. Pharm..

[64] Baucells M., Ferrer N., Gómez P., Lacort G., Roura M. (1993). Determination of caffeine in solid pharmaceutical samples by FTIR spectroscopy. Mikrochim. Acta.

[65] Imran M., Iqubal M.K., Imtiyaz K., Saleem S., Mittal S., Rizvi M.M.A., Ali J., Baboota S. (2020). Topical nanostructured lipid carrier gel of quercetin and resveratrol: Formulation, optimization, in vitro and ex vivo study for the treatment of skin cancer. Int. J. Pharm..

[66] Altamimi M.A., Elzayat E.M., Qamar W., Alshehri S.M., Sherif A.Y., Haq N., Shakeel F. (2019). Evaluation of the bioavailability of hydrocortisone when prepared as solid dispersion. Saudi Pharm. J. SPJ Off. Publ. Saudi Pharm. Soc..

[67] Sitkowski L., Stefaniak L., Nicol L., Martin M.L., Martin G.J., Webb G.A. (1995). Complete assignments of the 1H, 13C and 15N NMR spectra of caffeine. Spectrochim. Acta Part A Mol. Biomol. Spectrosc..

[68] Ivanova G., Simeonova M., Cabrita E.J., Rangel M. (2011). NMR insight into the supramolecular structure of daunorubicin loaded polymer nanoparticles. J. Phys. Chem. B.

[69] Heins A., McPhail D.B., Sokolowski T., Stöckmann H., Schwarz K. (2007). The location of phenolic antioxidants and radicals at interfaces determines their activity. Lipids.

[70] Ferreira L.E., Muniz B.V., Burga-Sánchez J., Volpato M.C., de Paula E., Rosa E.A., Groppo F.C. (2017). The effect of two drug delivery systems in ropivacaine cytotoxicity and cytokine release by human keratinocytes and fibroblasts. J. Pharm. Pharmacol..

[71] Chen R., Lin J., Hong J., Han D., Zhang A.D., Lan R., Fu L., Wu Z., Lin J., Zhang W. (2014). Potential toxicity of quercetin: The repression of mitochondrial copy number via decreased POLG expression and excessive TFAM expression in irradiated murine bone marrow. Toxicol. Rep..

[72] Pourtalebi Jahromi L., Ghazali M., Ashrafi H., Azadi A. (2020). A comparison of models for the analysis of the kinetics of drug release from PLGA-based nanoparticles. Heliyon.

[73] Baksi R., Singh D.P., Borse S.P., Rana R., Sharma V., Nivsarkar M. (2018). In vitro and in vivo anticancer efficacy potential of Quercetin loaded polymeric nanoparticles. Biomed. Pharmacother. Biomed. Pharmacother..

[74] Willson C. (2018). The clinical toxicology of caffeine: A review and case study. Toxicol. Rep..

[75] Arooj A., Rehman A.U., Iqbal M., Naz I., Alhodaib A., Ahmed N. (2023). Development of Adapalene Loaded Liposome Based Gel for Acne. Gels.

[76] Lau E.T., Giddings S.J., Mohammed S.G., Dubois P., Johnson S.K., Stanley R.A., Halley P.J., Steadman K.J. (2013). Encapsulation of hydrocortisone and mesalazine in zein microparticles. Pharmaceutics.

[77] Nadal J.M., Dos Anjos Camargo G., Novatski A., Macenhan W.R., Dias D.T., Barboza F.M., Lyra A., Roik J.R., Padilha de Paula J., Somer A. (2019). Adapalene-loaded poly(ε-caprolactone) microparticles: Physicochemical characterization and in vitro penetration by photoacoustic spectroscopy. PLoS ONE.

[78] Liang X.W., Xu Z.P., Grice J., Zvyagin A.V., Roberts M.S., Liu X. (2013). Penetration of nanoparticles into human skin. Curr. Pharm. Des..

